# Electron-injection-engineering induced dual-phase MoO_2.8_F_0.2_/MoO_2.4_F_0.6_ heterostructure for magnesium storage

**DOI:** 10.1093/nsr/nwae238

**Published:** 2024-07-11

**Authors:** Weixiao Wang, Fangyu Xiong, Shaohua Zhu, Mengyu Yan, Xiaobin Liao, Kesong Yu, Lianmeng Cui, Jinghui Chen, Junjun Wang, Ruoqi Lan, Jun Xie, Qinyou An, Liqiang Mai

**Affiliations:** State Key Laboratory of Advanced Technology for Materials Synthesis and Processing, Wuhan University of Technology, Wuhan 430070, China; State Key Laboratory of Advanced Technology for Materials Synthesis and Processing, Wuhan University of Technology, Wuhan 430070, China; State Key Laboratory of Advanced Technology for Materials Synthesis and Processing, Wuhan University of Technology, Wuhan 430070, China; State Key Laboratory of Advanced Technology for Materials Synthesis and Processing, Wuhan University of Technology, Wuhan 430070, China; State Key Laboratory of Advanced Technology for Materials Synthesis and Processing, Wuhan University of Technology, Wuhan 430070, China; State Key Laboratory of Advanced Technology for Materials Synthesis and Processing, Wuhan University of Technology, Wuhan 430070, China; State Key Laboratory of Advanced Technology for Materials Synthesis and Processing, Wuhan University of Technology, Wuhan 430070, China; State Key Laboratory of Advanced Technology for Materials Synthesis and Processing, Wuhan University of Technology, Wuhan 430070, China; State Key Laboratory of Advanced Technology for Materials Synthesis and Processing, Wuhan University of Technology, Wuhan 430070, China; State Key Laboratory of Advanced Technology for Materials Synthesis and Processing, Wuhan University of Technology, Wuhan 430070, China; State Key Laboratory of Silicate Materials for Architectures, Wuhan University of Technology, Wuhan 430070, China; State Key Laboratory of Advanced Technology for Materials Synthesis and Processing, Wuhan University of Technology, Wuhan 430070, China; Hubei Longzhong Laboratory, Wuhan University of Technology (Xiangyang Demonstration Zone), Xiangyang 441000, China; Hainan Institute, Wuhan University of Technology, Sanya 572000, China; State Key Laboratory of Advanced Technology for Materials Synthesis and Processing, Wuhan University of Technology, Wuhan 430070, China; Hubei Longzhong Laboratory, Wuhan University of Technology (Xiangyang Demonstration Zone), Xiangyang 441000, China; Hainan Institute, Wuhan University of Technology, Sanya 572000, China

**Keywords:** rechargeable magnesium batteries, electron injection strategy, dual-phase heterostructure, electronic conductivity, ionic diffusivity

## Abstract

Rechargeable magnesium batteries (RMBs) have received increased attention due to their high volumetric capacity and safety. Nevertheless, the sluggish diffusion kinetics of highly polarized Mg^2+^ in host lattices severely hinders the development of RMBs. Herein, we report an electron injection strategy for modulating the Mo 4d-orbital splitting manner and first fabricate a dual-phase MoO_2.8_F_0.2_/MoO_2.4_F_0.6_ heterostructure to accelerate Mg^2+^ diffusion. The electron injection strategy triggers weak Jahn–Teller distortion in MoO_6_ octahedra and reorganization of the Mo 4d-orbital, leading to a partial phase transition from orthorhombic phase MoO_2.8_F_0.2_ to cubic phase MoO_2.4_F_0.6_. As a result, the designed heterostructure generates a built-in electric field, simultaneously improving its electronic conductivity and ionic diffusivity by at least one order of magnitude compared to MoO_2.8_F_0.2_ and MoO_2.4_F_0.6_. Importantly, the assembled MoO_2.8_F_0.2_/MoO_2.4_F_0.6_//Mg full cell exhibits a remarkable reversible capacity of 172.5 mAh g^−1^ at 0.1 A g^−1^, pushing forward the orbital-scale manipulation for high-performance RMBs.

## Teaser

An electron injection strategy is proposed to modulate Mo 4d-orbital splitting manner and fabricate orthorhombic/cubic phase MoO_2.8_F_0.2_/MoO_2.4_F_0.6_ heterostructure for improving the magnesium storage performance.

## INTRODUCTION

Rechargeable magnesium batteries (RMBs) have great potential for the next-generation energy storage due to low cost, high volumetric capacity (3833 mAh cm^−3^), and dendrite-free formation [1–3]. However, strong electrostatic interactions of polarized Mg^2+^ and host lattice could cause sluggish electrochemical reaction kinetics in RMBs, which severely restricts their development [4,5]. Thus, seeking advanced cathode materials for realizing rapid ion and charge transfer is highly desirable to alleviate the aforementioned issue.

Recently, molybdenum-based oxides have been widely explored for energy storage applications due to their high theoretical capacity and abundant crystal structure [6]. Nevertheless, the widespread utilization of molybdenum oxides for RMBs has been restricted by their intrinsic characteristics, such as poor electrical conductivity and sluggish Mg^2+^ diffusivity [7]. Until now, extensive efforts have been devoted to overcome these weaknesses, such as enlarging interlayer spacing by preintercalation molecules or ions [8,9], shielding the strong polarization of Mg^2+^ by introducing H_2_O molecules [10], and reducing binding force by doping anions with higher polarizability [11]. Despite the Mg^2+^ migration barriers in metallic oxides in which materials can be significantly reduced, the ionic diffusion kinetics of full cells assembled with a Mg metal anode still remains a challenge in respect to the poor affinity of O^2−^ in the crystal frameworks and Mg^2+^. F^−^ doping can significantly reduce both the bandgap value and the Mg-ion migration barriers, which have been proved by theoretical calculation [12]. In our previous reports, F^−^ substitutional doping of molybdenum-based oxides improved the affinity of anion and Mg^2+^, thereby accelerating Mg^2+^ diffusion along three dimensional (3D) pathways in materials [13]. Unfortunately, the designed electrodes usually lead to an insufficient improvement in magnesium storage due to uncoordinated regulation of the electronic conductivity and ionic diffusivity of materials.

In order to realize the coordination of electronic conductivity and ionic diffusivity, some researchers pay attention to the built-in electric field (BIEF) in the heterostructure materials [14,15]. The BIEF could trigger charge redistribution behavior and regulate the electronic structure for achieving superior conductivity of heterostructure materials [16]. In addition, the built-in electric field could induce abundant ion adsorption and accelerate ion diffusion from a higher majority concentration to a lower majority concentration in the interfaces of heterostructures [17,18]. However, the strategy of constructing built-in electric fields gives a simple insight into the underlying regulatory mechanisms which limit the improvement of the effect of a BIEF [19]. In this regard, the deep understanding of constructing a BIEF has been regarded to be a nascent topic [20,21]. Therefore, developing a highly efficient and orbital-scale manipulation strategy to achieve the establishment of a BIEF is urgent but still remains challenging.

In this work, we propose an electron injection strategy to induce Mo 4d-orbital splitting manner modulation and fabricate an orthorhombic/cubic phase MoO_2.8_F_0.2_/MoO_2.4_F_0.6_ (o-c MoO_2.8_F_0.2_/MoO_2.4_F_0.6_) heterostructure to improve Mg storage performance. The electron injection strategy triggers a weak Jahn–Teller distortion in MoO_6_ octahedra and reorganization of the Mo 4d orbitals, leading to a partial phase transition (orthorhombic to cubic) for achieving a dual-phase MoO_2.8_F_0.2_/MoO_2.4_F_0.6_ heterostructure. The designed heterostructure possesses an abundant built-in electric field, which simultaneously enhances electron transfer and ion diffusion in the crystal frameworks. The Mott–Schottky analysis and X-ray absorption near-edge structure (XANES) analyses are employed to understand the generation and effect mechanism of the BIEF in the heterostructure. In addition, I-V tests, galvanostatic intermittent titration techniques (GITTs) and density functional theory (DFT) calculations were investigated to test the enhancement of the electronic conductivity and ionic diffusivity. As a result, the o-c MoO_2.8_F_0.2_/MoO_2.4_F_0.6_ electrode displays high reversible capacity (303.8 mAh g^−1^ at 0.1 A g^−1^) and excellent rate performance (154.1 mAh g^−1^ at 2 A g^−1^). Importantly, the assembled o-c MoO_2.8_F_0.2_/MoO_2.4_F_0.6_//Mg full cell exhibits a remarkable reversible capacity of 172.5 mAh g^−1^ at 0.1 A g^−1^. Briefly, our current work provides an efficient strategy for achieving coordinated regulation of electronic conductivity and ionic diffusivity in cathode materials.

## RESULTS AND DISCUSSION

**Figure 1.**
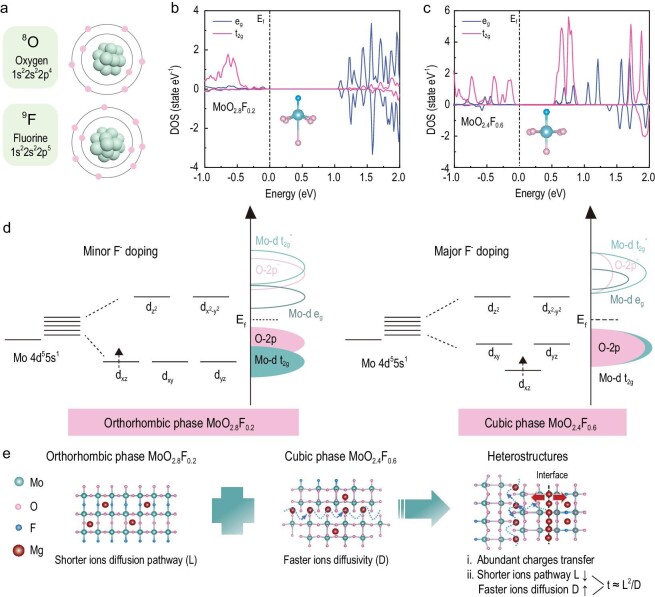
(a) The atomic structures of oxygen and fluorine. Calculated PDOS of (b) o-MoO_2.8_F_0.2_, and (c) c-MoO_2.4_F_0.6_. (d) Schematic diagram of the Mo 4d orbitals splitting manner. (e) Schematic illustration of Mg^2+^ diffusion in o-MoO_2.8_F_0.2_, c-MoO_2.4_F_0.6_, and o-c MoO_2.8_F_0.2_/MoO_2.4_F_0.6_ heterostructures.

Oxygen (O) and fluorine (F) are adjacently located in the same period (Period II) of the Periodical Table of Elements, which were selected to form the octahedral TMX_6_ (where ‘TM’ is ‘transition metal’, ‘X’ is ‘O or F’) with molybdenum for the study [22]. However, the difference in electronegativity and valence electron configuration (O: 2s^2^2p^4^, F: 2s^2^2p^5^) results in the injection of electrons and reorganization of the Mo 4d orbitals in the octahedral TMX_6_ when replacing the O element with an F element (Fig. 1a). The projected density of states (PDOS) calculations were performed to examine the local electron configuration of the MoO_3_, o-MoO_2.8_F_0.2_ and c-MoO_2.4_F_0.6_ (Fig. S1 in the online supplementary file, and Fig. 1b, c). The orthorhombic phase MoO_2.8_F_0.2_ (o-MoO_2.8_F_0.2_) reveals the asymmetric arrangement of Mo 4d orbital electrons, indicating the emergence of spin polarization after minor aliovalent F^−^ doping. The 4d orbit of o-MoO_2.8_F_0.2_ is similar to that of octahedral TMO_6_ and still retains the orthorhombic phase of MoO_3_. Nevertheless, the cubic phase MoO_2.4_F_0.6_ (c-MoO_2.4_F_0.6_) exhibits distinct spin polarization, which is attributed to the weak Jahn–Teller distortion in MoO_6_ octahedra after major aliovalent F^−^ doping. The crystal structure of MoO_3_, o-MoO_2.75_F_0.25_, and c-MoO_2.5_F_0.5_ are shown in Figs S2 and S3. Based on the crystal field theory, the Mo 4d orbital splitting manner of MoO_3_ in the quasi-octahedral field features the doubly degenerate states e_g_ orbitals (d_z^2^_ and d_x^2^__-__y^2^_) and triply degenerate states t_2__g_ orbitals (d_xz_, d_yz_, and d_xy_). Then, following the minor electron injection, the Mo 4d orbital splitting manner of o-MoO_2.8_F_0.2_ has not changed significantly, and the crystal phase remains the in the orthorhombic phase (Fig. 1d). After the major electron injection, major electrons spill over into the d_xz_ orbital and results in the downshift of the energy level of d_xz_ orbitals in the Mo 4d orbital splitting manner of c-MoO_2.4_F_0.6_. Therefore, the transformation of Mo 4d orbital splitting manner and crystal phase are transformed from the orthorhombic phase to the cubic phase. Conclusion, minor aliovalent F^−^ doping MoO_3_ (o-MoO_2.8_F_0.2_) introduces a spot of electrons and induces negligible lattice distortion. Besides, major aliovalent F^−^ doping MoO_3_ (c-MoO_2.4_F_0.6_) induces the reorganization of the Mo 4d orbitals and leads to significant lattice distortion. Compared with MoO_3_, o-MoO_2.8_F_0.2_ generates molybdenum vacancies for unlocking the inactive basal plane of the layered crystal structure. This architecture shortens ion diffusion length (L) along the b-axis and ac plane in the crystal frameworks. In addition, c-MoO_2.4_F_0.6_ activates the blocked crystal structure and increases ion diffusivity (D) in the materials. Therefore, the o-c MoO_2.8_F_0.2_/MoO_2.4_F_0.6_ heterostructure is delicately designed via integrating the o-MoO_2.8_F_0.2_ and c-MoO_2.4_F_0.6_, which could simultaneously reduce Mg^2+^ diffusion time (t) in two aspects (t ≈ L^2^/D) (Fig. 1e, Fig. S4). In addition, the abundant interfaces in MoO_2.8_F_0.2_/MoO_2.4_F_0.6_ heterostructures could trigger charge redistribution and regulate the electronic structure for achieving superior conductivity of heterostructure materials. Furthermore, the interfaces could induce abundant ion adsorption and accelerate ion diffusion. Based on the above analysis, the o-c MoO_2.8_F_0.2_/MoO_2.4_F_0.6_ heterostructure is delicately designed via the modulation of the aliovalent F^−^ doping in MoO_3_, with the specific control strategy and synthesis process being summarized in Figs S5–S7. For values of 29<R<79, single crystals of the orthorhombic phase MoO_2.8_F_0.2_ were prepared with the samples being characterized by a deep blue color. For values of 14<R<29, products consisting of both phases were prepared, including the orthorhombic phase MoO_2.8_F_0.2_ and cubic phases MoO_2.4_F_0.6_. However, until now, a one-step method makes it difficult to realize extremely dense o-c interfacial sites due to the asynchronous nucleation and growth of the two phases (Fig. S8). Therefore, the multi-step synthetic process of the orthorhombic phase and cubic phase MoO_2.8_F_0.2_/MoO_2.4_F_0.6_ heterostructure are implemented to realize an orderly layered nanostructure and dense o-c interfaces.

**Figure 2.**
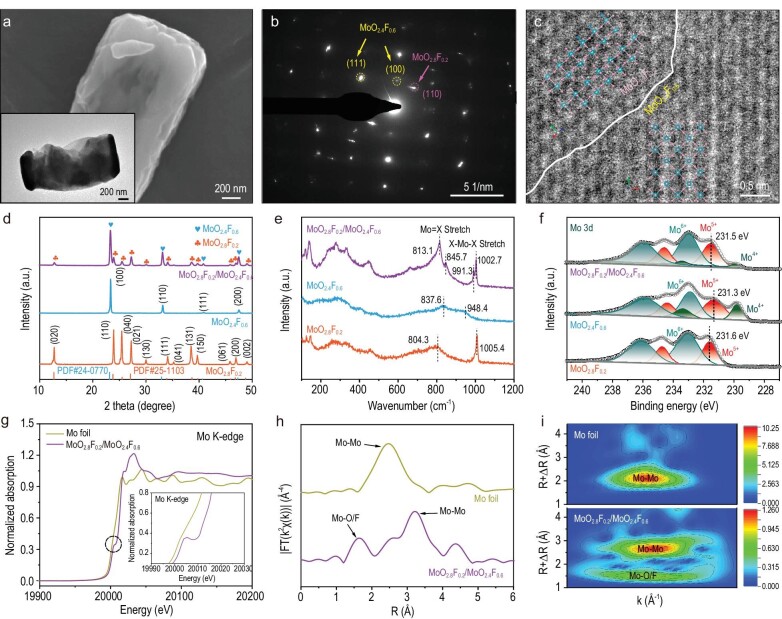
(a) SEM image and TEM image, (b) SAED pattern, and (c) HAADF-STEM image of o-c MoO_2.8_F_0.2_/MoO_2.4_F_0.6_ heterostructures. (d) XRD patterns, (e) Raman spectra, and (f) Mo 3d XPS spectra of o-MoO_2.8_F_0.2_, c-MoO_2.4_F_0.6_, and o-c MoO_2.4_F_0.6_/MoO_2.8_F_0.2_ heterostructures. (g) The normalized Mo K-edge XANES spectra, (h) FT-EXAFS spectra, and (i) WT-EXAFS spectra of o-c MoO_2.4_F_0.6_/MoO_2.8_F_0.2_ heterostructures and Mo foil.

The scanning electron microscopy (SEM) and transmission electron microscopy (TEM) images of the o-c MoO_2.8_F_0.2_/MoO_2.4_F_0.6_ heterostructure shows a layered nanostructure with the layers arranged in a neat and orderly manner (Fig. 2a, Fig. S9), which exhibits a more uniform morphology than o-c MoO_2.8_F_0.2_/MoO_2.4_F_0.6_ composites with synchronous synthesis process (Figs S10 and S11). The high-resolution TEM (HRTEM) image clearly uncovers the two-phase feature of the heterostructure (Fig. S12). The lattice fringe of ∼0.372 nm can be indexed to (110) planes of o-MoO_2.8_F_0.2_ materials, and another set of interplanar spacing ∼0.238 nm is ascribed to (111) plane of c-MoO_2.4_F_0.6_ materials. It's worth noting that there is some unambiguous lattice disorder in the o-c MoO_2.8_F_0.2_/MoO_2.4_F_0.6_ heterostructure, which is caused by the reorganization of the Mo 4d orbitals. In Fig. 2b, the selected-area electron diffraction (SAED) pattern exhibits two sets of diffraction spots, which further proves the coexistence of the cubic and orthorhombic phases. To visualize the real interfacial distribution, the high-angle annular dark field STEM image (HAADF-STEM) is shown in Fig. 2c. The heterostructure composes the orthorhombic phase MoO_2.8_F_0.2_ with distorted MoX_6_ octahedra sharing edges and corners and cubic phase MoO_2.4_F_0.6_ with regular MoX_6_ octahedra sharing corners. In addition, the HRTEM image by FIB treatment and in-depth Mo 3d XPS spectra distinctly reveal the two-phase feature of the heterostructure within the bulk phase of the active material particles (Figs S13 and S14). Moreover, SEM images and TEM images of MoO_3_, o-MoO_2.8_F_0.2_, and c-MoO_2.4_F_0.6_ are shown in Figs S15–S18. The corresponding EDS spectra confirm the proportion of the Mo, O, and F elements in o-MoO_2.8_F_0.2_ and c-MoO_2.4_F_0.6_ (Fig. S19). In addition, the distinct color of the samples is displayed in the photographs in Fig. S20. In Fig. 2d, X-ray diffraction (XRD) patterns reveal that the diffraction peaks of the o-c MoO_2.8_F_0.2_/MoO_2.4_F_0.6_ heterostructure match well with the orthorhombic phase MoO_2.8_F_0.2_ (Cmcm (63), JCPDS No. 25–0563) and cubic phase MoO_2.4_F_0.6_ (Pm-3 m (221), JCPDS No. 24–0770), which verifies the coexistence of o-MoO_2.8_F_0.2_ and c-MoO_2.4_F_0.6_ in the heterostructure [23,24]. The as-synthesized o-c MoO_2.8_F_0.2_/MoO_2.4_F_0.6_ heterostructure has been analyzed by X-ray diffraction (XRD) patterns and their Rietveld refinement (Fig. S21), in which the ratio of the orthorhombic phase MoO_2.8_F_0.2_ to the cubic phase MoO_2.4_F_0.6_ in the heterostructure is 1:1. In addition, the ratio of o-MoO_2.8_F_0.2_ and c-MoO_2.4_F_0.6_ as 8:2 (denoted by MoO_2.8_F_0.2_/MoO_2.4_F_0.6_–1) and 2:8 (denoted by MoO_2.8_F_0.2_/MoO_2.4_F_0.6_–2) have been prepared to understand the correlations between phase ratios and electrochemical performance.

The Raman spectra of o-MoO_2.8_F_0.2_, c-MoO_2.4_F_0.6_, and o-c MoO_2.8_F_0.2_/MoO_2.4_F_0.6_ heterostructures are displayed in Fig. 2e. The peaks at 804.3 and 1005.4 cm^−1^ represent the Mo=X stretching vibrations and X−Mo−X stretching vibrations (where X represents O or F) of o-MoO_2.8_F_0.2_ materials, respectively (Table S1). Besides, the characteristic peaks of c-MoO_2.4_F_0.6_ can be found at 837.6 and 948.4 cm^−1^. In addition, two characteristic peaks of the o-c MoO_2.8_F_0.2_/MoO_2.4_F_0.6_ heterostructure appear at 813.1 and 845.7 cm^−1^, corresponding to the Mo=X symmetric stretching vibrations of the o-MoO_2.8_F_0.2_ and c-MoO_2.4_F_0.6_ materials. Two characteristic peaks of the heterostructure appear at 991.3 and 1002.7 cm^−1^, which signifies the Mo=X asymmetric stretching vibrations of the o-MoO_2.8_F_0.2_ and c-MoO_2.4_F_0.6_ materials [25]. Interestingly, compared with o-MoO_2.8_F_0.2_ and c-MoO_2.4_F_0.6_, the peaks of the o-c MoO_2.8_F_0.2_/MoO_2.4_F_0.6_ heterostructure show a slight shift, which is related to the change in the bond angles/lengths of the MoX_6_ octahedron during the different number of electrons injected (Fig. S22) [26]. In addition, the XRD pattern and Raman spectrum of MoO_3_ are displayed in Fig. S23. The chemical valence states of samples are explored by X-ray photoelectron spectroscopy (XPS). In Fig. S24, the survey spectra indicate the existence of Mo, O, and F elements in o-MoO_2.8_F_0.2_, c-MoO_2.4_F_0.6_, and o-c MoO_2.8_F_0.2_/MoO_2.4_F_0.6_ heterostructures. From the Mo 3d spectra (Fig. 2f), the peaks at 236.1 and 233.1 eV are assigned to Mo 3d_3/2_ and Mo 3d_5/2_ signals of Mo^6+^, and the peaks at 234.7 and 231.6 eV are attributed to Mo 3d_3/2_ and Mo 3d_5/2_ signals of Mo^5+^. In addition, the Mo 3d XPS spectrum of c-MoO_2.4_F_0.6_ shows extra peaks at 233.4 and 229.8 eV corresponding to Mo 3d_3/2_ and Mo 3d_5/2_ signals of Mo^4+^, which originate from the successful accomplishment of the major F^−^ doping. In the o-c MoO_2.8_F_0.2_/MoO_2.4_F_0.6_ heterostructure, the Mo 3d spectrum can be fitted to three pairs of peaks that are related to Mo^6+^ (236.0 and 233.0 eV), Mo^5+^ (234.6 and 231.5 eV), and Mo^4+^ (233.5 and 229.9 eV) [27]. Remarkably, the binding energy of the o-c MoO_2.8_F_0.2_/MoO_2.4_F_0.6_ heterostructure is apparently higher by ∼0.1–0.3 eV than that in c-MoO_2.4_F_0.6_, while the binding energy of the heterostructure is slightly lower compared to o-MoO_2.8_F_0.2_. The opposite shift of Mo 3d confirms the distribution of opposite charges through the interface in o-c MoO_2.8_F_0.2_/MoO_2.4_F_0.6_ heterostructures [28]. In Fig. S25, the F 1s XPS spectrum of the o-c MoO_2.8_F_0.2_/MoO_2.4_F_0.6_ heterostructure is also red-shifted and blue-shifted compared to the o-MoO_2.8_F_0.2_ and c-MoO_2.4_F_0.6_, respectively, which is associated with the charge transfer between o-MoO_2.8_F_0.2_ and c-MoO_2.4_F_0.6_. As a contrast, XPS spectra of pure MoO_3_ are displayed in Fig. S26. Furthermore, we have performed electron paramagnetic resonance (EPR) spectra to identify the existence of molybdenum vacancies in o-MoO_2.8_F_0.2_ and c-MoO_2.4_F_0.6_ materials. As shown in Fig. S27, compared with the pristine MoO_3_ sample, the o-MoO_2.8_F_0.2_ sample exhibits an intense EPR signal at g = 2.002 which could be attributed to the presence of unpaired electrons in Mo species following minor F^−^ doping. Furthermore, the characteristic peak of the Mo−O/F dangling bond for the c-MoO_2.4_F_0.6_ sample is much stronger than that of the o-MoO_2.8_F_0.2_ sample, demonstrating a significantly increased concentration of molybdenum defects following major F^−^ doping. These cationic vacancies are conducive to unlocking the inactive basal plane of the layered crystal structure and triggering shallow impurity levels in the energy band, giving rise to fast Mg^2+^ diffusion and electron transport during charge/discharge processes. Moreover, the Mo K-edge X-ray absorption near-edge structure (XANES) analyses of the o-c MoO_2.8_F_0.2_/MoO_2.4_F_0.6_ heterostructure was implemented (Fig. 2g). The distinct pre-edge peak of the o-c MoO_2.8_F_0.2_/MoO_2.4_F_0.6_ heterostructure exhibits an asymmetrical structure, which may be ascribed to the hybridization of orbitals [29]. The first-derivative of the XANES spectra is shown in Fig. S28. The Fourier-transform (FT) and wavelet-transform (WT) EXAFS spectra (Fig. 2h, i) confirm the existence of both Mo-O/F and Mo-Mo coordination [30]. The peaks of Mo-Mo in Mo foil are concentrated at 2.47 Å, and the peaks of Mo-O/F and Mo-Mo in the o-c MoO_2.8_F_0.2_/MoO_2.4_F_0.6_ heterostructure correspond to 1.63 Å and 3.21 Å, respectively [31].

**Figure 3.**
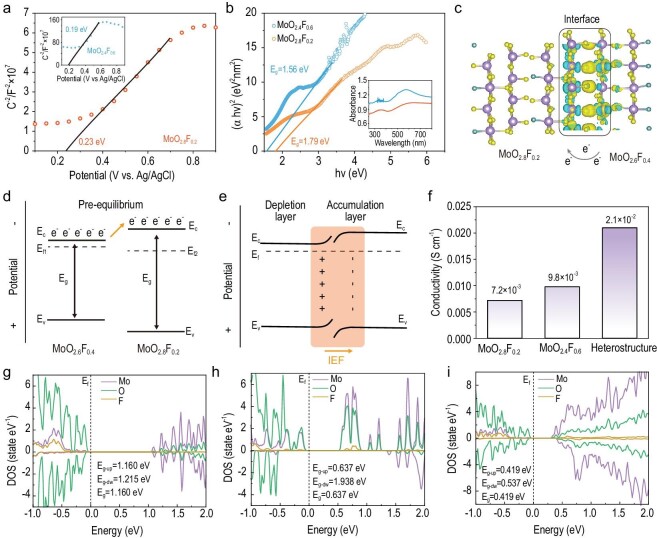
(a) Mott-Schottky curves of c-MoO_2.4_F_0.6_ and o-MoO_2.8_F_0.2_. (b) UV-vis diffuse reflectance spectra of o-MoO_2.8_F_0.2_ and c-MoO_2.4_F_0.6_. (c) Charge density difference image of the o-c MoO_2.8_F_0.2_/MoO_2.4_F_0.6_ heterostructures. (d and e) Schematic diagrams of bond alignment of o-c MoO_2.8_F_0.2_/MoO_2.4_F_0.6_ heterostructures. (f) Electrical conductivity of o-MoO_2.8_F_0.2_, c-MoO_2.4_F_0.6_ and o-c MoO_2.8_F_0.2_/MoO_2.4_F_0.6_ heterostructures. Calculated TDOS of (g) o-MoO_2.8_F_0.2_, (h) c-MoO_2.4_F_0.6_, and (i) o-c MoO_2.8_F_0.2_/MoO_2.4_F_0.6_ heterostructures.

To gain more detailed information about the band gap (E_g_), valence band (E_vb_), and conductive band (E_cb_) levels of samples, related tests are conducted and shown in Fig. 3. The Boltzmann distribution and Gauss’ law reveals the electron distributions in the space charge region, which are associated with the BIEF at the interface of the o-c MoO_2.8_F_0.2_/MoO_2.4_F_0.6_ heterostructure [32]. Therefore, the Mott–Schottky equation can be obtained through Poisson's equation [33]:


\begin{eqnarray*}
\frac{1}{{{{C}^2}}} = \frac{2}{{\varepsilon {{\varepsilon }_0}{{A}^2}e{{N}_D}}}\left( {V - {{V}_{fb}} - \frac{{{{k}_B}T}}{e}} \right),
\end{eqnarray*}

where *C, A, e, V, k_B_* and *T* are the interfacial capacitance area, charge, voltage, Boltzmann's constant, and absolute temperature, respectively. In Fig. 3a and Fig. S29, the Mott–Schottky plots of o-MoO_2.8_F_0.2_ and c-MoO_2.4_F_0.6_ derived from the plots of Log Z against potential exhibit a positive slope, which is a typical n-type semiconductor [34]. In addition, the flat band potentials (E_fb_) of materials can be confirmed from the Mott–Schottky curves. The E_fb_ of o-MoO_2.8_F_0.2_ and c-MoO_2.4_F_0.6_ are 0.23 and 0.19 eV vs. Ag/AgCl, which are equal to 0.43 and 0.39 eV vs. normal hydrogen electrode (NHE), respectively. Notably, as an n-type semiconductor, the E_fb_ is usually ∼0.1–0.3 eV (defined as 0.20 eV) more positive than their conductive band position (E_cb_) [35]. Therefore, the E_cb_ of o-MoO_2.8_F_0.2_ and c-MoO_2.4_F_0.6_ can be approximately estimated as 0.23 and 0.19 eV, respectively. To disclose the band gap (E_g_) of materials, the UV−vis spectra are displayed in Fig. 3b. Obviously, the o-MoO_2.8_F_0.2_ and c-MoO_2.4_F_0.6_ exhibit strong absorption at ∼400 nm wavelength, and the corresponding E_g_ values are 1.79 and 1.56 eV, respectively, which are smaller than the E_g_ value of pure MoO_3_ (3.03 eV) (Fig. S30). Theoretically, the valence band (E_vb_) is relevant to the band gap (E_g_) and conductive band (E_cb_), which can be described as [36]:


\begin{eqnarray*}
{\mathrm{E_g}} = {\mathrm{E_{\rm vb}}} - {\mathrm{E_{\rm cb}}}.
\end{eqnarray*}

Thereby, the E_vb_ of o-MoO_2.8_F_0.2_ and c-MoO_2.4_F_0.6_ can be calculated as 2.02 and 1.75 eV, respectively. Besides, the calculated work functions (ϕ) of o-MoO_2.8_F_0.2_ and c-MoO_2.4_F_0.6_ are 4.67 and 4.59 eV, respectively (Figs S31, and S32). The E_vb_, E_g_, and E_cb_ of o-MoO_2.8_F_0.2_ and c-MoO_2.4_F_0.6_ are summarized in Table S2. Accordingly, significant charge transfer from c-MoO_2.4_F_0.6_ to o-MoO_2.8_F_0.2_ and imbalanced charge distribution are further displayed in the calculated results (Fig. 3c). Once o-MoO_2.8_F_0.2_ and c-MoO_2.4_F_0.6_ are in intimate contact, the charge redistribution will spontaneously occur near the interfaces until their Fermi levels reach equilibrium (Fig. 3d and e). As a result, the electrons and holes will be accumulated on the o-MoO_2.8_F_0.2_ and c-MoO_2.4_F_0.6_ sides, respectively, which forms a certain built-in electric field for providing a continuous electron flow. As proof, the electrical conductivity of the o-MoO_2.8_F_0.2_, c-MoO_2.4_F_0.6_, and o-c MoO_2.8_F_0.2_/MoO_2.4_F_0.6_ heterostructures are displayed, and their calculated conductivities are 7.2 × 10^−3^, 9.8 × 10^−3^, and 2.1 × 10^−2^ S cm^−1^, respectively (Fig. 3f). The result reveals that the BIEF in the o-c MoO_2.8_F_0.2_/MoO_2.4_F_0.6_ heterostructure significantly enhances conductivity by orders of magnitude. To deeper understand the role of heterojunctions in enhancing electronic conductivity and ionic diffusivity, the total density of states (TDOS) calculations and the finite element simulation were carried out. As shown in Fig. 3g–i, the total density of states (TDOS) of o-MoO_2.8_F_0.2_, c-MoO_2.4_F_0.6_, and o-c MoO_2.8_F_0.2_/MoO_2.4_F_0.6_ heterostructures reveal that F^−^ doping related states have emerged in the bandgap of those materials. Notably, the o-c MoO_2.8_F_0.2_/MoO_2.4_F_0.6_ heterostructure displays a narrow band gap in the vicinity of the Fermi level (E_f_), which generates more unpaired electrons and electronic conduction owing to the built-in electric field in the heterostructure.

To clarify the merits of the heterostructure, the obtained materials were evaluated in a magnesium half-cell, in which the activated carbon cloth (AC) with high surface area and abundant active sites are used as counter electrodes. The AC electrode delivers reversible capacities of 0.14 mAh at 0.1 A g^−1^ in the window between −0.15 and 0.15 V vs. Ag/AgCl (Fig. S33), which can provide enough capacity and charges to balance the Mg^2+^ intercalation process in the o-c MoO_2.8_F_0.2_/MoO_2.4_F_0.6_ electrode [37,38]. In the half-cell, the magnesium ions derived from the electrolyte are inserted/extracted in the cathode and both anions (TFSI^−^) and cations (Mg^2+^) from the electrolyte are reversibly adsorbed/desorbed in the activated carbon cloth (AC) anode. The anions adsorb while the cations desorb on the surface of the AC anode during the discharge process, and *vice versa* during the charge process (Fig. S34). The reaction process of the o-c MoO_2.8_F_0.2_/MoO_2.4_F_0.6_//AC cells can be formulated as follows:

In the discharge process:


(1) \begin{eqnarray*}
{\mathrm{Cathode}}{:}&&{\mathrm{\ Mo}}{{{\mathrm{O}}}_{2.8}}{{{\mathrm{F}}}_{0.2}}/{\mathrm{Mo}}{{{\mathrm{O}}}_{2.4}}{{{\mathrm{F}}}_{0.6}} + x{\mathrm{M}}{{{\mathrm{g}}}^{2 + }}\\
&& +\, 2x{{{\mathrm{e}}}^ - } \to {\mathrm{M}}{{{\mathrm{g}}}_x}{\mathrm{Mo}}{{{\mathrm{O}}}_{2.8}}{{{\mathrm{F}}}_{0.2}}/{\mathrm{Mo}}{{{\mathrm{O}}}_{2.4}}{{{\mathrm{F}}}_{0.6}}\\
\end{eqnarray*}

(2) \begin{eqnarray*}
{\mathrm{Anode}}{:}&& {{\left( {\mathrm{AC\, containing}\,\,{\it y}{\rm M{{g}^{2 + }}}} \right)}_{\mathrm{surface}}}\\
&&\quad +\, 2\left( {x - y} \right){\mathrm{TFS}{{I}^ - } - 2{\it x}{{e}^{ - }}}\\
&& \to {{\left( {\mathrm{AC\, containing}\, 2\left( {{\it x} - {\it y}} \right){\mathrm{TFS{{I}^ - }}}} \right)}_{\mathrm{surface}}}\\
&&\quad +\, {\mathrm{{\it y}M{{g}^{2 + }}}}
\end{eqnarray*}

In the charge process:


(3) \begin{eqnarray*}
{\mathrm{Cathode}}{:}&&{\mathrm{M}}{{g}_x}{\mathrm{Mo}}{{{\mathrm{O}}}_{2.8}}{{{\mathrm{F}}}_{0.2}}/{\mathrm{Mo}}{{{\mathrm{O}}}_{2.4}}{{{\mathrm{F}}}_{0.6}} - {\rm 2{\it x}{{e}^ - }}\\
&&\quad \!\!\to {\mathrm{Mo}}{{{\mathrm{O}}}_{2.8}}{{{\mathrm{F}}}_{0.2}}/{\mathrm{Mo}}{{{\mathrm{O}}}_{2.4}}{{{\mathrm{F}}}_{0.6}} + x{\mathrm{M}}{{g}^{2 + }}\!\!\\
\end{eqnarray*}

(4) \begin{eqnarray*}
{\mathrm{Anode}}{:}&&{{\left( {{\mathrm{AC}}\,\,\,{\mathrm{containing\, 2}}\left( {x - y} \right){\mathrm{TFS}}{{{\mathrm{I}}}^{\mathrm{ - }}}} \right)}_{{\mathrm{surface}}}}\\
&&\quad +\, y{\mathrm{M}}{{g}^{2 + }} + {\rm 2{\it x}{{e}^ - }} \\
&& \to {{\left( {{\mathrm{AC\, containing}}\,\,{\mathrm{{\it y}M}}{{g}^{2 + }}} \right)}_{{\mathrm{surface}}}}\\
&&\quad +\, 2\left( {x - y} \right){\mathrm{TFS}}{{{\mathrm{I}}}^ - }
\end{eqnarray*}

**Figure 4.**
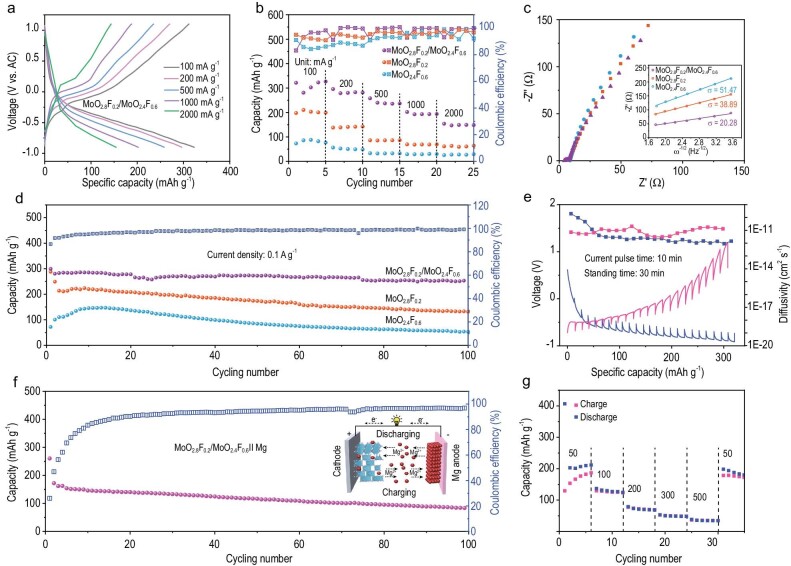
(a) GCD curves at different currents of o-c MoO_2.8_F_0.2_/MoO_2.4_F_0.6_ electrode. (b) Rate capability, (c) Nyquist plots, and (d) cycling performances at 0.1 A g^−1^ of o-MoO_2.8_F_0.2_, c-MoO_2.4_F_0.6_ and o-c MoO_2.8_F_0.2_/MoO_2.4_F_0.6_ electrodes. (e) GITT curves of o-c MoO_2.8_F_0.2_/MoO_2.4_F_0.6_ electrode. (f) Cycling performance and (g) rate performance of the assembled o-c MoO_2.8_F_0.2_/MoO_2.4_F_0.6/_/Mg full cell.

In addition, the charge/discharge processes of the MoO_2.8_F_0.2_/MoO_2.4_F_0.6_//AC cells are schematically illustrated in Fig. S35. Cyclic voltammetry (CV) curves of o-c MoO_2.8_F_0.2_/MoO_2.4_F_0.6_ electrodes are measured for the initial cycles at a scan rate of 0.2 mV s^−1^. As depicted in Fig. S36, a pair of obvious redox reaction peaks are attributed to the insertion and extraction of Mg^2+^ in the o-c MoO_2.8_F_0.2_/MoO_2.4_F_0.6_ electrode. These peaks are well overlapped over the three cycles, demonstrating the good electrochemical reversibility of the heterostructure. In Fig. 4a, the galvanostatic charge-discharge (GCD) curves of o-c MoO_2.8_F_0.2_/MoO_2.4_F_0.6_ electrode were performed at various current densities, which displays well-defined voltage platforms. Compared with pure o-MoO_2.8_F_0.2_ and c-MoO_2.4_F_0.6_ electrodes, the o-c MoO_2.8_F_0.2_/MoO_2.4_F_0.6_ electrode delivers improved reversible capacities of 321.7, 295.9, 258, 202.1, and 154.1 mAh g^−1^ at the current density 0.1, 0.2, 0.5, 1, and 2 A g^−1^, respectively (Fig. 4b). Noteworthy, the relatively high Coulombic efficiency of the o-c MoO_2.8_F_0.2_/MoO_2.4_F_0.6_ heterostructure is attributed to the enhancement of electronic conductivity and ionic diffusivity by at least an order of magnitude. To better understand the impact of the o-c MoO_2.8_F_0.2_/MoO_2.4_F_0.6_ heterostructure on the Mg^2+^ diffusion behaviors, the Nyquist plots and fitted results of electrodes are shown in Fig. 4c. The o-c MoO_2.8_F_0.2_/MoO_2.4_F_0.6_ electrode exhibits smaller Warburg coefficients (σ = 20.28) than that of the o-MoO_2.8_F_0.2_ electrode (σ = 38.89) and c-MoO_2.4_F_0.6_ electrode (σ = 51.47) [39,40], which further proves the efficient diffusion kinetics of Mg^2+^ in the o-c MoO_2.8_F_0.2_/MoO_2.4_F_0.6_ electrode. Moreover, the o-c MoO_2.8_F_0.2_/MoO_2.4_F_0.6_ electrode delivers a reversible capacity of 303.8 mAh g^−1^ at 0.1 A g^−1^, which is much higher than that of pure o-MoO_2.8_F_0.2_ (249.3 mAh g^−1^) and c-MoO_2.4_F_0.6_ (100.6 mAh g^−1^) (Fig. 4d). Therefore, Mg^2+^ prefers to insert/extract in the o-c MoO_2.8_F_0.2_/MoO_2.4_F_0.6_, which may be attributed to the abundant phase boundary. Furthermore, the ion diffusion coefficient (D) of electrodes was investigated by GITT tests. The D of the o-c MoO_2.8_F_0.2_/MoO_2.4_F_0.6_ electrode is 9.12 × 10^−13^ to 1.99 × 10^−10^ cm^2^ s^−1^, which is much higher than pure o-MoO_2.8_F_0.2_ electrode and c-MoO_2.4_F_0.6_ electrode (Fig. 4e; Figs S37, S38, and S39), revealing the efficient ion transfer enabled by the dual-phase heterostructure. In addition, the D values of the MoO_3_ electrode is 2.27 × 10^−14^ to 2.5 × 10^−12^ cm^2^ s^−1^ (Fig. S40), which is much lower than o-MoO_2.8_F_0.2_ electrode (1.41 × 10^−13^ to 4.25 × 10^−12^ cm^2^ s^−1^) and c-MoO_2.4_F_0.6_ electrode (5.55 × 10^−14^ to 4.15 × 10^−12^ cm^2^ s^−1^), showing that the substitution of fluorine for oxygen could activate low-valent redox-active transition metals or create more cationic vacancies.

Further investigation of the correlations between the phase ratios of MoO_2.8_F_0.2_/MoO_2.4_F_0.6_ heterostructure and electrochemical performance are shown in Fig. S41. Although the cation defects in the materials significantly improve the Mg^2+^ diffusion, the interfacical content in the MoO_2.8_F_0.2_/MoO_2.4_F_0.6_ heterostructure plays an even more crucial role in accelerating ion diffusion kinetics. Therefore, the D of MoO_2.8_F_0.2_/MoO_2.4_F_0.6_–1 and MoO_2.8_F_0.2_/MoO_2.4_F_0.6_–2 electrodes ranges from 8.94 × 10^−13^ to 2.32 × 10^−11^ cm^2^ s^−1^, which is notably lower compared to the o-c MoO_2.8_F_0.2_/MoO_2.4_F_0.6_ electrode (4.58 × 10^−13^ to 1.48 × 10^−11^ cm^2^ s^−1^), indicating sluggish ion diffusion kinetics in the other phase ratios. In addition, the MoO_2.8_F_0.2_/MoO_2.4_F_0.6_–1 and MoO_2.8_F_0.2_/MoO_2.4_F_0.6_–2 electrodes exhibit a low capacity and poor cycling stability. The D values of the o-c MoO_2.8_F_0.2_/MoO_2.4_F_0.6_ electrode is superior to the majority of previously reported molybdenum-based oxide electrodes (Table S3). The CV curves of the o-c MoO_2.8_F_0.2_/MoO_2.4_F_0.6_ electrode at different scan rates are displayed in Fig. S42. The b-values of the oxidation/reduction peaks are assessed at 0.88 and 0.86, respectively, demonstrating the electrochemical storage behavior contributed by both capacitive and diffusion behavior. To prove the enhancement of the cathode-electrolyte compatibility of the heterostructure in magnesium full cells, we assembled o-c MoO_2.8_F_0.2_/MoO_2.4_F_0.6_//Mg, MoO_2.8_F_0.2_//Mg, and MoO_2.4_F_0.6_//Mg full cells. In magnesium full cells, the MTB electrolyte was used due to its ability to achieve reversible deposition and dissolution of magnesium (Fig. S43). Impressively, the GCD curves of o-c MoO_2.8_F_0.2_/MoO_2.4_F_0.6_//Mg full cells were performed at 0.1 A g^−1^ (Fig. S44), which displays relatively little polarization and high reversible capacity of 172.5 mAh g^−1^ (Fig. 4f). The low initial Coulombic efficiency of a full cell is attributed to the thermodynamic instability of a Mg/organic electrolyte interface and the electrochemical conditioning process of MTB electrolyte during the initial Mg^2+^ deposition/dissolution process. In Fig. 4g, the rate performance of the cell was determined at different current densities and the corresponding reversible capacities are 203.3, 132.1, 73.6, 50.3, and 35.9 mAh g^−1^, respectively. Furthermore, a reversible capacity of 194.1 mAh g^−1^ was easily restored at 0.05 A g^−1^, indicating the favorable rate tolerance capability of the cell. As a contrast, the cycling performances and rate performances of o-MoO_2.8_F_0.2_//Mg and c-MoO_2.4_F_0.6_//Mg are displayed in Figs S45 and S46, which are inferior to the o-c MoO_2.8_F_0.2_/MoO_2.4_F_0.6_//Mg cell. In addition, the magnesium storage property of the o-c MoO_2.8_F_0.2_/MoO_2.4_F_0.6_//Mg cell is also superior to the majority of previously reported molybdenum-based materials (Table S4). In addition, the simple Mg(TFSI)_2_/AN and Mg(TFSI)_2_/DME electrolyte was used in the magnesium full cells system (Figs S47 and S48). However, these electrolytes can not support the operation of magnesium full cells due to the Mg metal passivating in polar aprotic solvents and the incomplete dissociation of magnesium salts.

**Figure 5.**
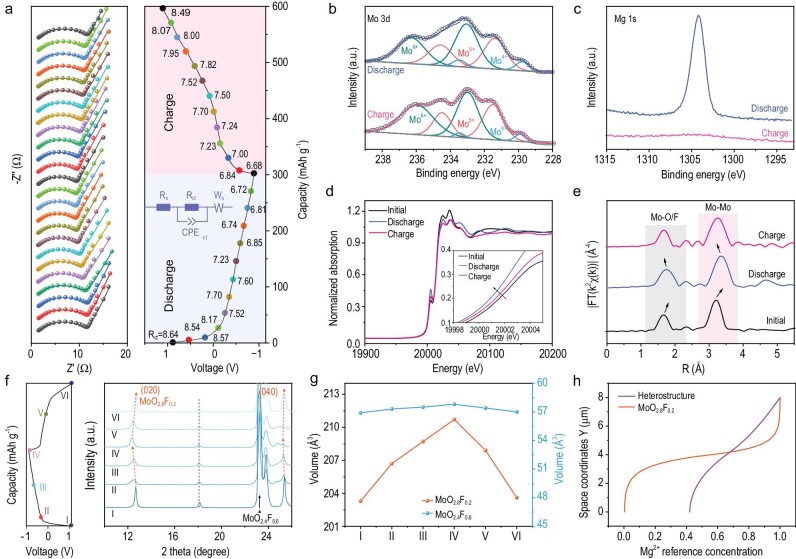
(a) The *in-situ* Nyquist plots and the corresponding fitting parameter (R_ct_) of the o-c MoO_2.8_F_0.2_/MoO_2.4_F_0.6_ electrode at various discharged/charged states. The *ex-situ* (b) Mo 3d XPS spectra, (c) Mg 1s XPS spectra, (d) normalized Mo K-edge XANES spectra, (e) FT-EXAFS spectra of the cell at discharged and charged states. (f) The GCD profiles and *ex-situ* XRD patterns, and (g) the volume changes of o-c MoO_2.8_F_0.2_/MoO_2.4_F_0.6_ electrode at different voltage states. (h) The relative concentrations of magnesium ions in o-MoO_2.8_F_0.2_ and o-c MoO_2.8_F_0.2_/MoO_2.4_F_0.6_ heterostructures.

To further evaluate the kinetic in depth, *in situ* EIS measurement and the corresponding detailed fitting data were employed at various charge/discharge states of o-c MoO_2.8_F_0.2_/MoO_2.4_F_0.6_ electrode (Fig. 5a). When gradually discharging, the electrode shows a continual decrease of resistances due to the formation of Mo^4+^ and rich contact of the hetero-interface during the Mg^2+^ insertion process. When gradually charging, the continual increase of resistances is attributed to the reversible Mg^2+^ extraction process and exhibits excellent interfacial stability within the whole cycle. As shown in Fig. S49, STEM-EDS mapping images display the uniform dispersion of the Mg element, which reveals the Mg^2+^ insertion during the discharge process. In contrast, only the fuzzy Mg element outline can be observed within the charged o-c MoO_2.8_F_0.2_/MoO_2.4_F_0.6_ electrode. In addition, *ex-situ* Mo 3d XPS spectra were performed to examine the valence state of Mo in the o-c MoO_2.8_F_0.2_/MoO_2.4_F_0.6_ electrode during the charge/discharge process (Fig. 5b). After full discharging to 0.01 V, the Mo^4+^ content is significantly improved, which derives from the reduction of Mo^6+^ and Mo^5+^. After full charging to 2.0 V, parts of Mo^4+^ in the o-c MoO_2.8_F_0.2_/MoO_2.4_F_0.6_ electrode are returned to Mo^6+^ and Mo^5+^, confirming the reversible evolution of the Mo valence state during the discharge/charge process. Besides, when discharged to 0.1 V, a sharp Mg 1s peak is observed, which indicates the surface adsorption and intercalation of Mg^2+^ in the o-c MoO_2.8_F_0.2_/MoO_2.4_F_0.6_ electrode (Fig. 5c). When charged to 2.0 V, the Mg signals almost disappeared, implying the reversible insertion/extraction of Mg^2+^. As depicted in Fig. S50, the peaks of Mo^6+^, Mo^5+^, and Mo^4+^ remain almost unchanged after 100 cycles, suggesting the high stability of the cell. In addition, the o-c MoO_2.8_F_0.2_/MoO_2.4_F_0.6_ heterostructure shows the layered and uniform nanostructure as well as the morphology are consistent with the SEM observation before 100 cycles (Fig. S51), which indicates the excellent stability of these materials. Moreover, the time-of-flight secondary-ion mass spectrometry (TOF-SIMS) of o-MoO_2.8_F_0.2_, c-MoO_2.4_F_0.6_ and o-c MoO_2.8_F_0.2_/MoO_2.4_F_0.6_ cathodes are implemented to explore the stability of high electronic conductivity o-c MoO_2.8_F_0.2_/MoO_2.4_F_0.6_ in chloride-containing electrolyte systems (Fig. S52). The presence of CH_2_O^−^ and Cl^−^ ionic fragments is correlated with the decomposition of both the solvent and chloride in the electrolyte. With the prolonged sputtering time, the signal intensity of CH_2_O^−^ and Cl^−^ ionic fragments decreases and stabilizes at the same time in o-MoO_2.8_F_0.2_, c-MoO_2.4_F_0.6_, and o-c MoO_2.8_F_0.2_/MoO_2.4_F_0.6_ electrodes, which means that the high electronic conductivity o-c MoO_2.8_F_0.2_/MoO_2.4_F_0.6_ could not worsen the continuous breakdown of the electrolyte, especially in chloride-containing electrolyte systems. In addition, the TOF-SIMS 3D views and depth profiles of the Mg^+^ ionic fragments in the o-c MoO_2.8_F_0.2_/MoO_2.4_F_0.6_ electrode is shown in Fig. S53. The results prove that an abundant amount of Mg^2+^ and a minimal amount of MgCl^+^ are intercalated into the o-c MoO_2.8_F_0.2_/MoO_2.4_F_0.6_ electrode during the discharge/charge processes, while a substantial amount of MgCl^+^ is absorbed on the surface of the electrode. As a further proof, a thin CEI layer in o-c MoO_2.8_F_0.2_/MoO_2.4_F_0.6_ cathodes with a thickness of ∼6.5 nm is observed in the HRTEM images (Fig. S54). To better understand the changes in valence states and charge transfer of the o-c MoO_2.8_F_0.2_/MoO_2.4_F_0.6_ electrode during magnesiation/demagnesiation, the Mo K-edge XANES analyses were measured. During the discharge process, the Mo edge shifts toward lower energy with the slight reduction of Mo^6+^ and Mo^5+^, and the white-line intensity decreases with the lattice expansion of the o-c MoO_2.8_F_0.2_/MoO_2.4_F_0.6_ electrode (Fig. 5d). The corresponding radical distribution of R-space are provided by Fourier transform of Mo K-edge XANES spectra. As shown in Fig. 5e, the average Mo-O/Mo-Mo bond slightly elongates and is shortened during the insertion and extraction of Mg^2+^, confirming the reversible evolution of valence state and lattice structure of the o-c MoO_2.8_F_0.2_/MoO_2.4_F_0.6_ electrode during the discharge/charge process. In addition, the heterostructure maintains structural stability during battery operation without irreversible phase transitions and significant volume changes. The *ex-situ* XRD patterns and variations of lattice volume of o-c MoO_2.8_F_0.2_/MoO_2.4_F_0.6_ at different voltage states were performed based on an AC anode (Fig. 5f, g). During the discharge and charging process, the (020) and (040) diffraction peaks of o-MoO_2.8_F_0.2_ shift and return, and the characteristic peaks of c-MoO_2.4_F_0.6_ show almost no change, indicating a highly reversible Mg^2+^ insertion/extraction process of the o-c MoO_2.8_F_0.2_/MoO_2.4_F_0.6_ electrode. To gain insights into the intrinsic properties of phases affecting ionic transfer kinetics, we conducted investigations into Mg^2+^ migration pathways within the orthorhombic and cubic phase structures using bond valence site energy (BVSE) calculations (Fig. S55). Visibly, the orthorhombic phase MoO_2.8_F_0.2_ provides intermittent and narrow zigzag paths for Mg^2+^ migration along the ac plane directions. Differently, the cubic phase MoO_2.4_F_0.6_ possesses successive and broad Mg^2+^ migration pathways along the 3D (a, b, and c-axis) directions. Impressively, the designed o-c MoO_2.8_F_0.2_/MoO_2.4_F_0.6_ heterostructure could trigger the reversible ion insertion/extraction and accelerate ion diffusion for achieving superior magnesium storage. To further prove the Mg^2+^ diffusion kinetics of the o-c MoO_2.8_F_0.2_/MoO_2.4_F_0.6_ heterostructure, the finite element simulation of Mg^2+^ concentration distribution for o-MoO_2.8_F_0.2_ and o-c MoO_2.8_F_0.2_/MoO_2.4_F_0.6_ heterostructures were simulated and analyzed (Fig. S56), in which the o-c MoO_2.8_F_0.2_/MoO_2.4_F_0.6_ heterostructure has a more uniform Mg^2+^ concentration and faster Mg^2+^ flux distribution compared to the o-MoO_2.8_F_0.2_ and c-MoO_2.4_F_0.6_. Figure 5h clearly and prominently displays the curves of relative concentration of magnesium ions in the structural materials, which suggests that the o-c MoO_2.8_F_0.2_/MoO_2.4_F_0.6_ heterostructure can achieve fast and efficient ion diffusion during the charge and discharge process. Besides, we summarized the comparison of the o-MoO_2.8_F_0.2_, c-MoO_2.4_F_0.6_, and o-c MoO_2.8_F_0.2_/MoO_2.4_F_0.6_ electrodes during the Mg^2+^ insertion/extraction process.

## CONCLUSIONS

To summarize, we innovatively designed the o-c MoO_2.8_F_0.2_/MoO_2.4_F_0.6_ heterostructure by an electron injection strategy to simultaneously enhance electronic conductivity and ionic diffusivity in RMBs. The electron injection strategy induces weak Jahn–Teller distortion in MoO_6_ octahedra and Mo 4d-orbital splitting, leading to a partial phase transition from orthorhombic MoO_2.8_F_0.2_ to cubic MoO_2.4_F_0.6_. The tailored dual-phase MoO_2.8_F_0.2_/MoO_2.4_F_0.6_ heterostructure triggers the built-in electric field, which shortens ion diffusion length (L) and activates ion diffusivity (D) in the crystal frameworks to reduce Mg^2+^ diffusion time in two aspects (t ≈ L^2^/D). In addition, the BIEF induces charge redistribution to decrease the band gap of the electronic structure. As a result, compared to pure o-MoO_2.8_F_0.2_ and c-MoO_2.4_F_0.6_, the electronic conductivity and ionic diffusion coefficient of o-c MoO_2.8_F_0.2_/MoO_2.4_F_0.6_ is improved by at least one order of magnitude. Impressively, the electrode demonstrates a respectable reversible capacity (303.8 mAh g^−1^ at 0.1 A g^−1^) and an excellent rate performance (154.1 mAh g^−1^ at 2 A g^−1^). Even after being assembled with a Mg anode, the full cell can provide a high specific capacitance of 172.5 mAh g^−1^ at 0.1 A g^−1^, exhibiting great potential for practical application. This work would offer a meaningful insight to simultaneously improve charge transfer and ion diffusion in the crystal frameworks for superior magnesium storage.

## METHODS

### Preparation of o-MoO_2.8_F_0.2_ materials

In a typical process, 3.6 g α-MoO_3_ powders and 0.06 g Mo powders were mixed in 37 mL DI water. Then, 6 mL HF (wt. 48%) was added to the above mixture and magnetically stirred for 1 h. Subsequently, the solution was added into a 50 mL Teflon-lined stainless-steel autoclave and kept at 200°C for 20 h. After cooling down, the blue products were collected by centrifugation with DI water and dried at 60°C for 12 h.

### Preparation of the o-c MoO_2.8_F_0.2_/MoO_2.4_F_0.6_ heterostructure

Specifically, 1.8 g of obtained MoO_2.8_F_0.2_ powders and 0.06 g Mo powders were dissolved into 37 mL DI water. Afterward, 6 mL HF (wt. 48%) was added to the above solution and magnetically stirred for 1 h. Then, the solution was directly moved to a 50 mL Teflon-lined stainless-steel autoclave and heated at 200°C for 20 h. Last, the deep blue products were centrifuged with DI water and dried at 60°C for 12 h. By varying the mole ratio (R) of MoO_3_ to Mo power in the reaction mixtures, different phase ratios of the heterostructure can be obtained, including the ratio of o-MoO_2.8_F_0.2_ and c-MoO_2.4_F_0.6_ as 8:2 (denoted by MoO_2.8_F_0.2_/MoO_2.4_F_0.6_–1) and 2:8 (denoted by MoO_2.8_F_0.2_/MoO_2.4_F_0.6_–2).

## Supplementary Material

nwae238_Supplemental_File
