# Supplementary material for: Electron-injection-engineering induced dual-phase MoO2.8F0.2/MoO2.4F0.6 heterostructure for magnesium storage
Source: Natl Sci Rev. 2024 Jul 11;11(8):nwae238. doi: 10.1093/nsr/nwae238 (PMC11312365; doi:10.1093/nsr/nwae238)
Supplement: nwae238_Supplemental_File [file nwae238_supplemental_file.pdf]

Supporting Information

Electron-injection-engineering induced dual-phase $MoO_{2.8}F_{0.2}/MoO_{2.4}F_{0.6}$ heterostructure for magnesium storage

Weixiao Wang,[1] Fangyu Xiong,[1] Shaohua Zhu,[1] Mengyu Yan,[1] Xiaobin Liao,[1] Kesong Yu,[1] Lianmeng Cui,[1] Jinghui Chen,[1] Junjun Wang,[1] Ruoqi Lan,[1] Jun Xie,[3] Qinyou An,[1,2,3] and Liqiang Mai,[1,2,3*]*

[1] State Key Laboratory of Advanced Technology for Materials Synthesis and Processing, Wuhan University of Technology, Wuhan 430070, China

2 Hubei Longzhong Laboratory, Wuhan University of Technology (Xiangyang Demonstration Zone), Xiangyang 441000, China.

3 Hainan Institute, Wuhan University of Technology, Sanya 572000, China.

4 State Key Laboratory of Silicate Materials for Architectures, Wuhan University of Technology, Wuhan 430070, China

E-mail: anqinyou86@whut.edu.cn; mlq518@whut.edu.cn

Materials Characterization

The morphology and microstructure of the materials were characterized by scanning electron microscope (SEM, JEOL-7100F) and transmission electron microscope (TEM, JEM-2100F/Titan G2 60–300). The crystal structures were identified by X-ray diffractometer (XRD, Bruker AXS D8) with Cu Kα radiation (λ=1.5406 Å). X-ray photoelectron spectroscopy (XPS) spectra were tested to analyze the chemical valence of the materials using Kratos AXIS SUPRA XPS instrument. The Raman spectra were recorded by Horiba LabRAM HR Evolution. The conductivity was investigated by four-point probe instrument (RM3000+, Jandel Engineering, UK). The Mott–Schottky plots were measured using a three-electrode system, in which the material was the working electrode, platinum wire was the opposite electrode, Ag/AgCl was the reference electrode, and 2M Na_2SO_4 was the electrolyte solution. UV-Vis diffuse reflectance spectra of the materials were displayed by Lambda 750 S.

Electrochemical measurements

The electrochemical performance was performed in CR2016 coin cells. The active materials, acetylene black and polyvinylidene fluoride (PVDF) were mixed with a weight ratio of 7:2:1 and dispersed in N-methyl pyrrolidone (NMP) solution. Then, the prepared slurry was casted on the carbon paper (thickness 0.05 mm, Alfa Aesar) and dried at 60 ℃ for 12 h, the working electrode was achieved. In half cell system, the activated carbon cloth (AC) was used as the counter electrode, and the immersion potential was calculated as 2.4 V vs. Mg/Mg^{2+}. 0.3 M magnesium bis (trifluoromethane sulfonyl) imide ($Mg (TFSI)_2$) in acetonitrile (AN) was used as the electrolyte. In the AC electrode side, the AC has a high specific surface area (ca. 2000 $m^2\ g^{-1}$), which conducts sufficient charge storage through electric double-layer capacitance and meets the requirement of complete charge balance during Mg^{2+} insertion. In the cathode side, the $MoO_{2.8}F_{0.2}/MoO_{2.4}F_{0.6}$ is capable of reversible Mg^{2+} insertion/extraction from its framework structure. In full cell system, the Mg metal was used as the anode, and MTB was

used as the electrolyte, in which 1.290 g $Mg(CF_3SO_3)_2$, 0.762 g $MgCl_2$, and 1.067 g $AlCl_3$ was dissolved in 20 ml of 1,2-dimethoxyethane (DME). The galvanostatic discharge-charge (GCD) and the galvanostatic intermittenttitration technique (GITT) measurements were carried out on the multichannel battery testing system (LAND CT2001A). The cyclic voltammetry (CV) and electrochemical impedance spectra (EIS) measurements were tested on a BioLogic science instruments.

DFT calculations

The density functional theory calculations were performed via the projector augmented plane-wave method, and implemented in the Vienna ab initio simulation package.[1] The generalzied gradient approximation proposed by Perdew, Burke, and Ernzerhof is applied for the exchange-correlation potential.[2] The long range van der Waals interaction is expressed by the DFT-D3 approach.[3] The cut-off energy for plane wave is set to 500 eV. The energy criterion is set to 10^{-5} eV in iterative solution of the Kohn-Sham equation. For the Brillouin zone integration, k-mesh of 5×2×5, 6×4×6, 4×4×1 for bulk $MoO_{2.75}F_{0.25}$ with Cmcm symmetry, bulk $MoO_{2.5}F_{0.5}$ with Pm-3m symmetry, and $MoO_{2.75}F_{0.25}/MoO_{2.5}F_{0.5}$ heterostructure, respectively. For the heterostructure of the $MoO_{2.75}F_{0.25}/MoO_{2.5}F_{0.5}$, a vacuum layer of 15 Å is added perpendicular to the sheet to avoid artificial interaction between periodic images. A Hubbard-U term is employed to treat the strongly correlated of the d-electrons of Mo atoms, the value of the effective Hubbard-U parameter, U_{eff} = 6.3 eV.[4] All the structures are fully relaxed until the residual forces on the atoms have declined to less than 0.02 eV/Å.

COMSOL Multiphysics 5.6 with the implemented finite element solver was used to study the Mg^{2+} concentration and Mg^{2+} flux distributions in the o-$MoO_{2.8}F_{0.2}$ and o-c $MoO_{2.8}F_{0.2}/MoO_{2.4}F_{0.6}$ heterostructures. The two models were calculated in a 2D region of 8×5 μm^2. The Mg^{2+} concentration was 4 M, and the Mg^{2+} diffusion coefficients in the o-$MoO_{2.8}F_{0.2}$ and o-c $MoO_{2.8}F_{0.2}/MoO_{2.4}F_{0.6}$ heterostructures were 1.0×10^{-14} and 1.4×10^{-15} m^2 s^{-1}, respectively. Finite element simulations were performed to study the von Mises stress distribution by applying the same constraint condition on the surface.

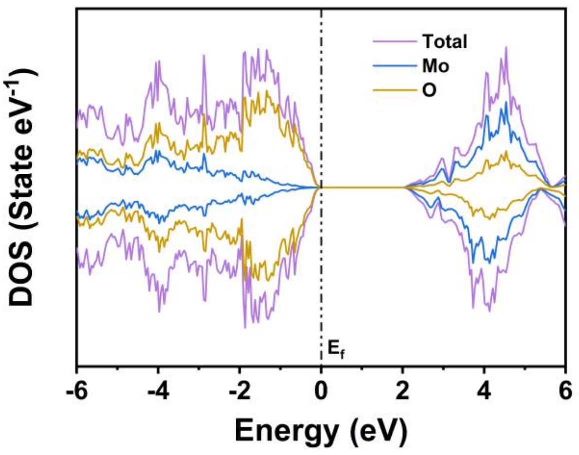

Figure S1. Calculated total density of states (TDOS) and partial density of states (PDOS) of MoO_3.

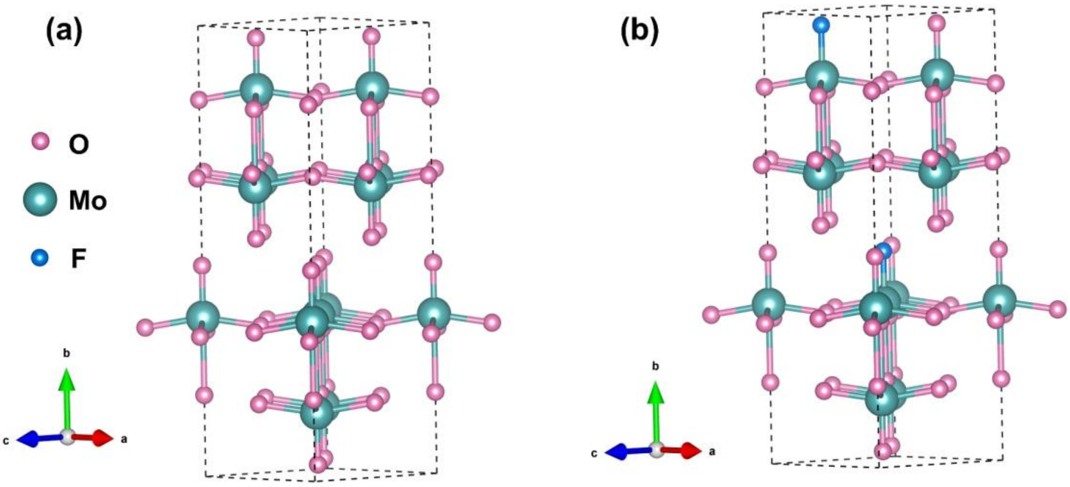

Figure S2. The crystal structure of the (a) MoO_3 and (b) $MoO_{2.75}F_{0.25}$ structure with orthorhombic phase Cmcm symmetry.

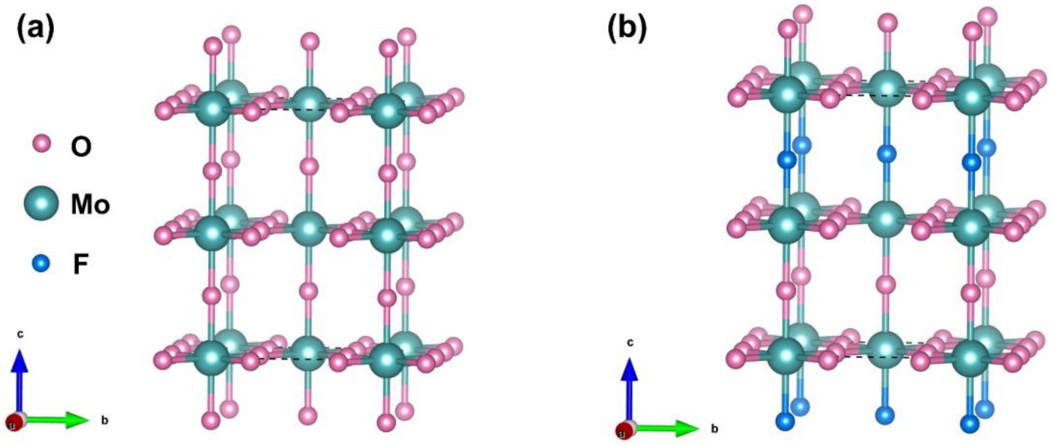

Figure S3. The crystal structure of the (a) MoO_3 and (b) $MoO_{2.5}F_{0.5}$ structure with cubic phase Pm3m symmetry.

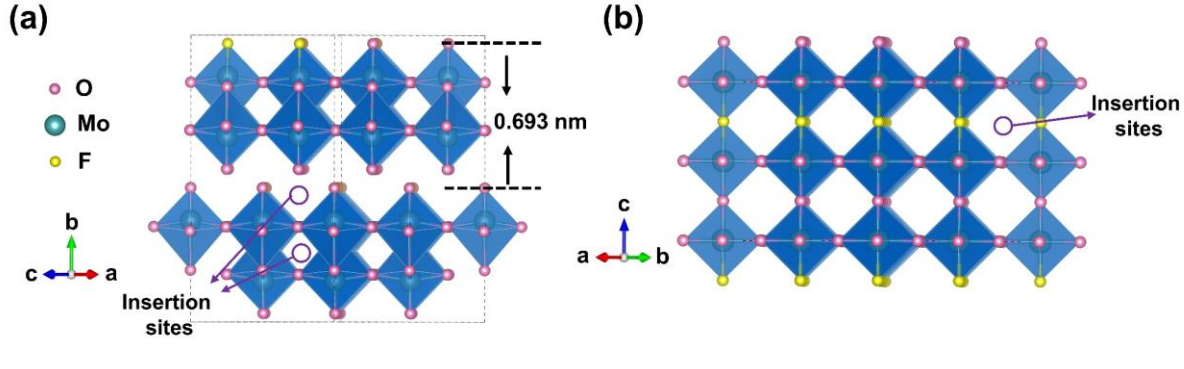

Figure S4. The insertion sites of Mg^{2+} of orthorhombic phase $MoO_{2.75}F_{0.25}$ and cubic phase $MoO_{2.5}F_{0.5}$.

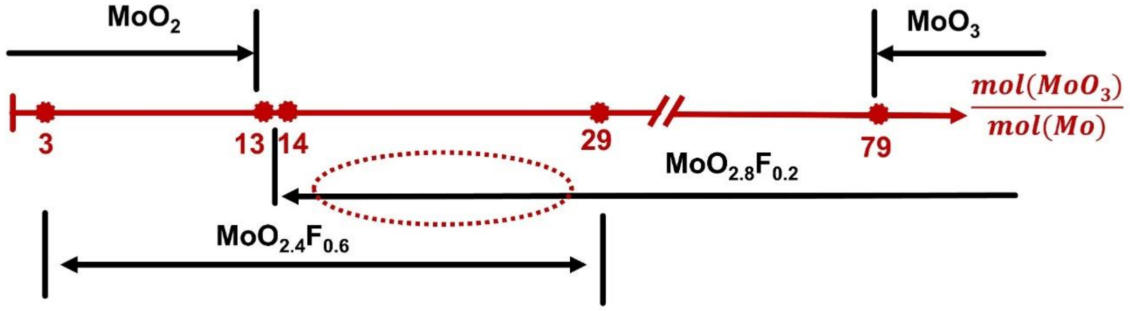

Figure S5. Different phase transition of the product affected by mole ratio of MoO_3 and Mo powers.

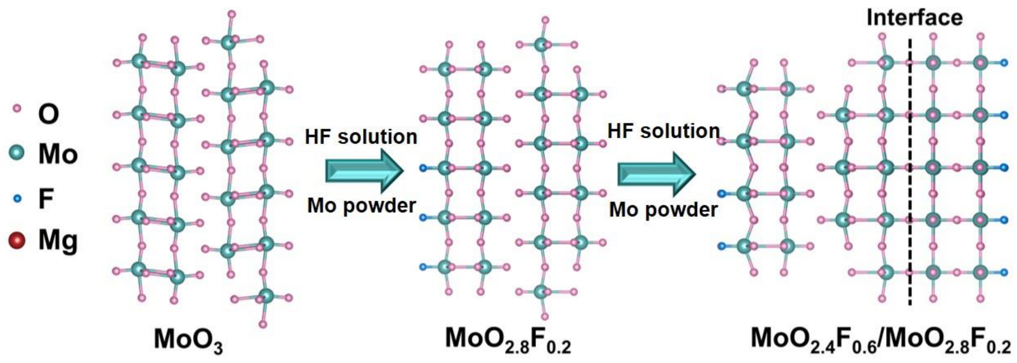

Figure S6. Schematic illustration of synthesis process of o-c $MoO_{2.8}F_{0.2}/MoO_{2.4}F_{0.6}$ heterostructures.

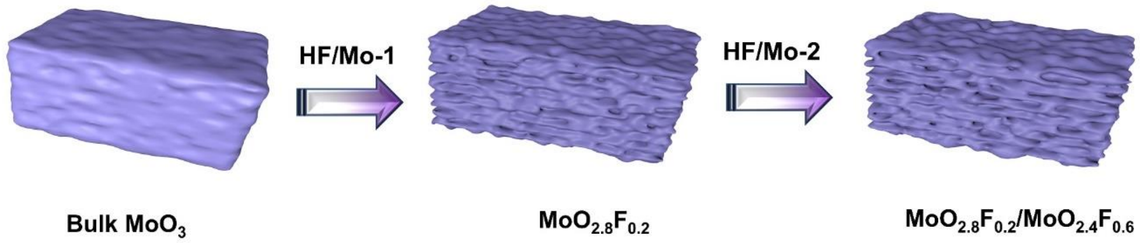

Figure S7. Schematic illustration of the asynchronous synthesis process of the o-c $MoO_{2.8}F_{0.2}/MoO_{2.4}F_{0.6}$ heterostructures.

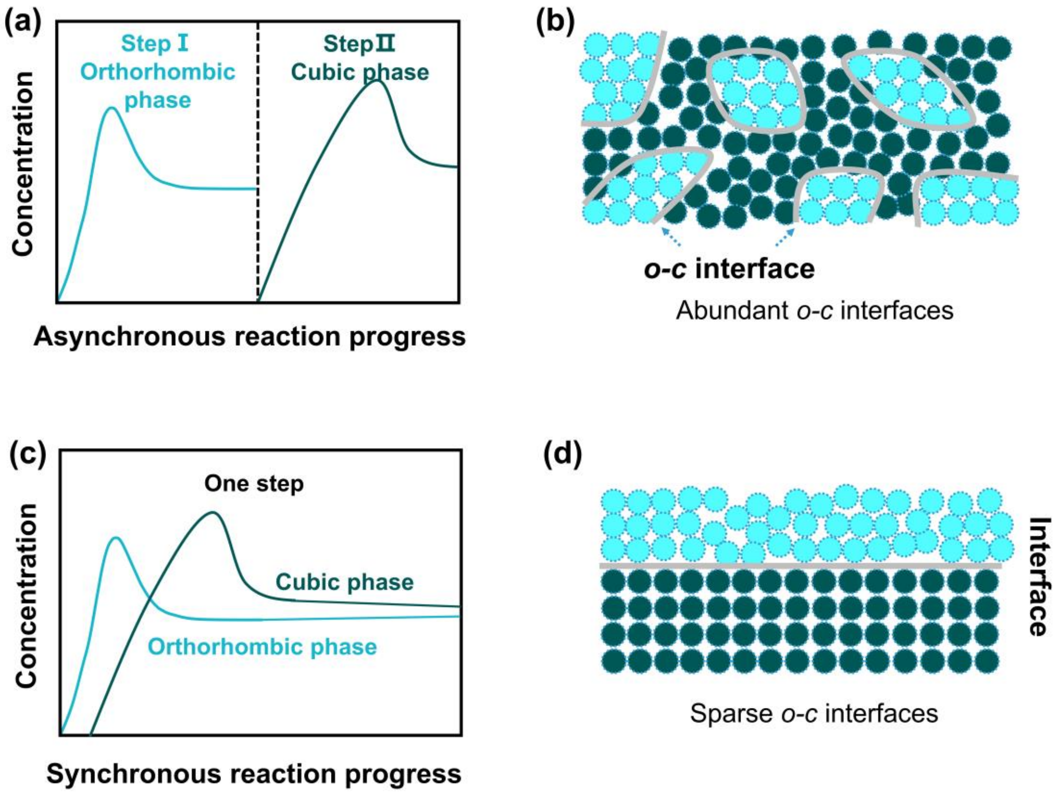

Figure S8. Schematic of the o-c $MoO_{2.8}F_{0.2}/MoO_{2.4}F_{0.6}$ heterostructures synthesis. (a, b) Asynchronous synthesis process, and (c, d) Synchronous synthesis process.

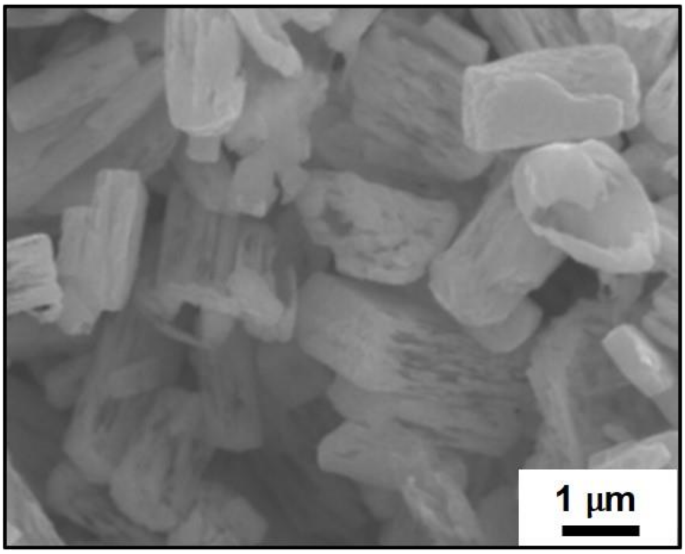

Figure S9. SEM image of o-c $MoO_{2.8}F_{0.2}/MoO_{2.4}F_{0.6}$ heterostructures.

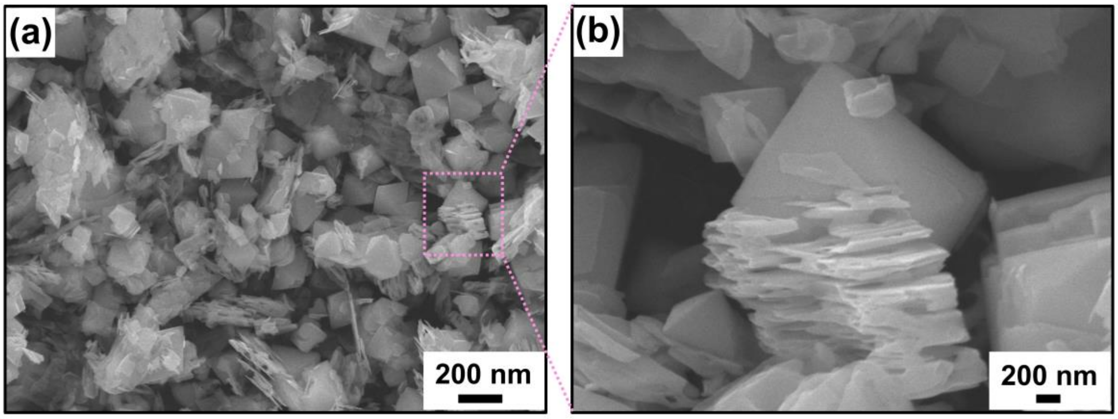

Figure S10. SEM images of o-c $MoO_{2.8}F_{0.2}/MoO_{2.4}F_{0.6}$ composite materials with synchronous synthesis process.

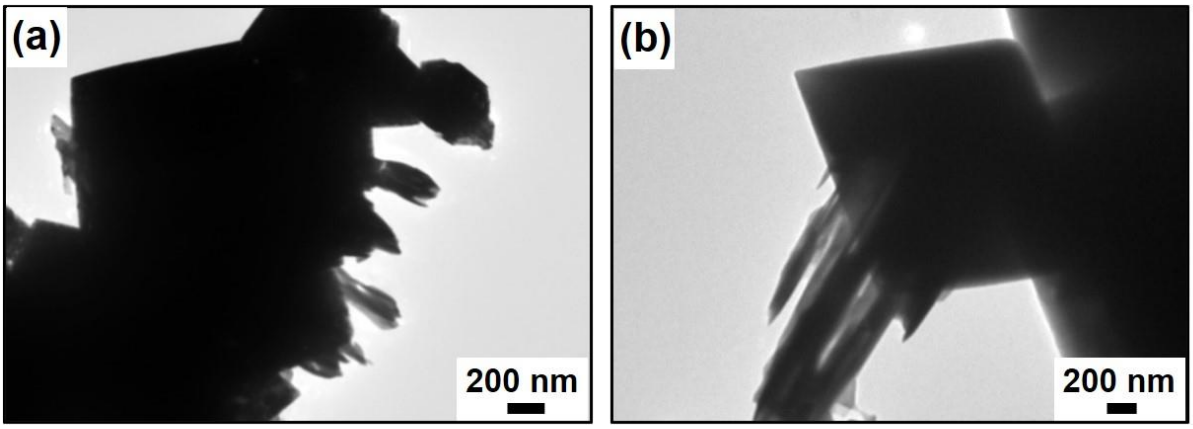

Figure S11. (a, b) TEM images of o-c $MoO_{2.8}F_{0.2}/MoO_{2.4}F_{0.6}$ composite materials with synchronous synthesis process.

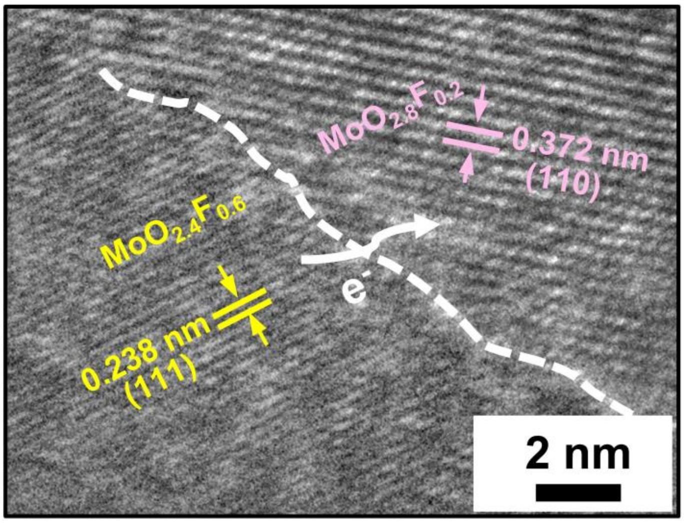

Figure S12. HRTEM image of o-c $MoO_{2.8}F_{0.2}/MoO_{2.4}F_{0.6}$ heterostructures.

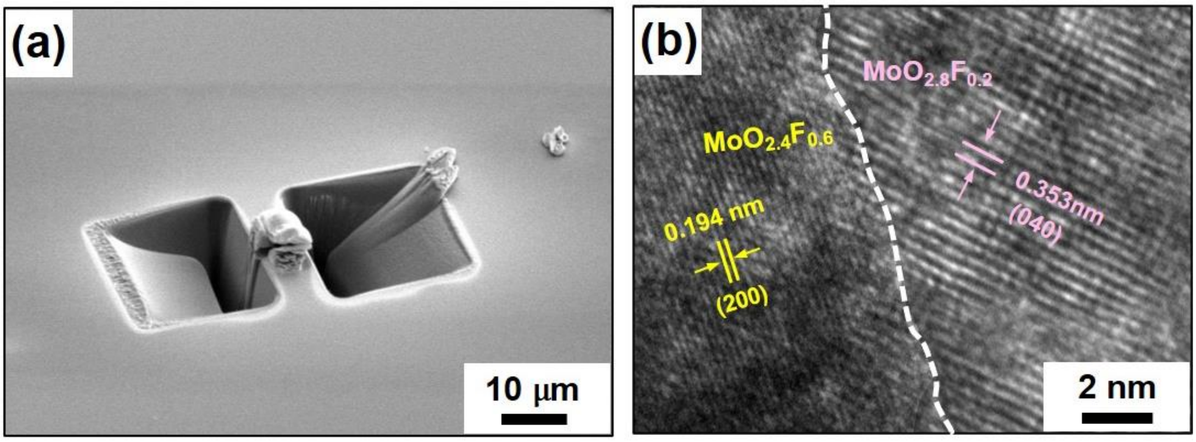

Figure S13. (a) FIBs treatment image, and (b) HRTEM image of o-c $MoO_{2.8}F_{0.2}/MoO_{2.4}F_{0.6}$ heterostructures.

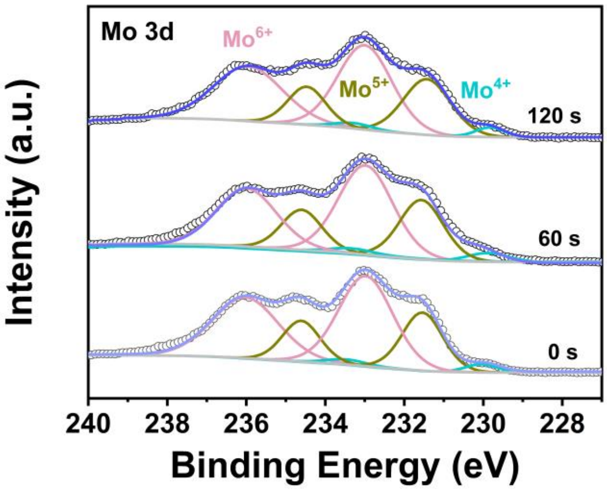

Figure S14. In-depth Mo 3d XPS spectra of $MoO_{2.8}F_{0.2}/MoO_{2.4}F_{0.6}$ electrodes.

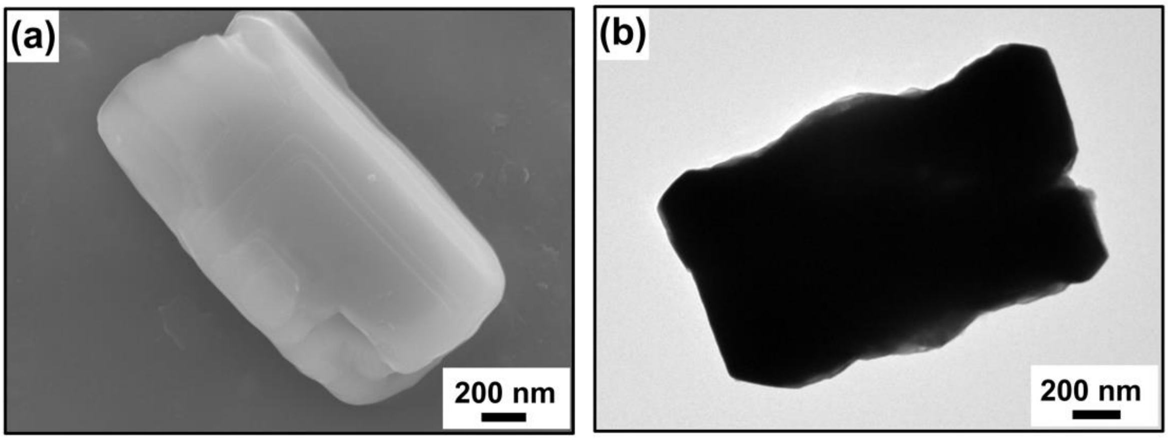

Figure S15. (a) SEM images and (b) TEM images of MoO_3.

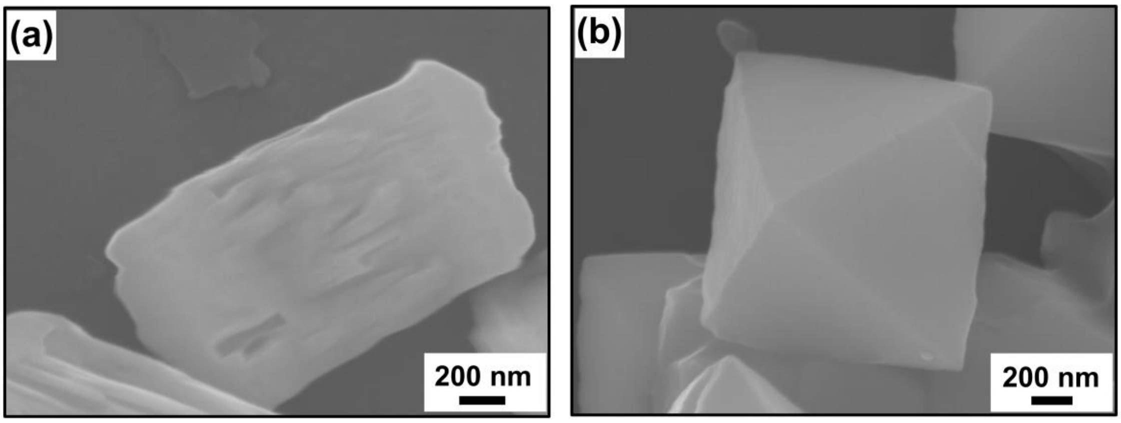

Figure S16. SEM images of (a) o-$MoO_{2.8}F_{0.2}$ and (b) c-$MoO_{2.4}F_{0.6}$.

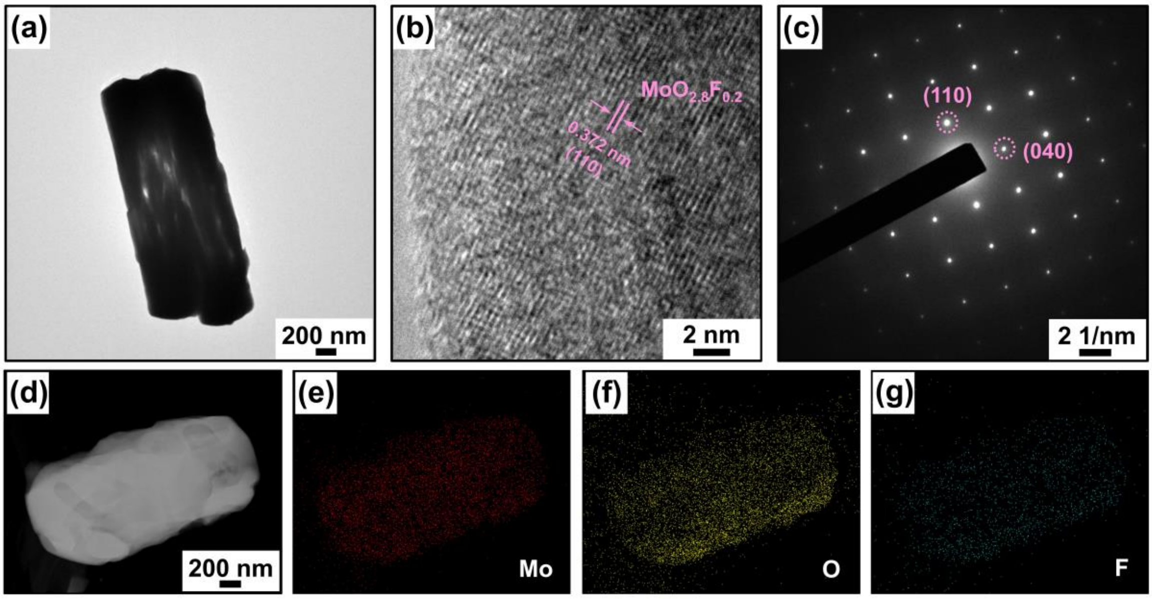

Figure S17. (a) TEM images, (b) HRTEM image, (c) SAED pattern, and (d) EDS mapping images of o-$MoO_{2.8}F_{0.2}$.

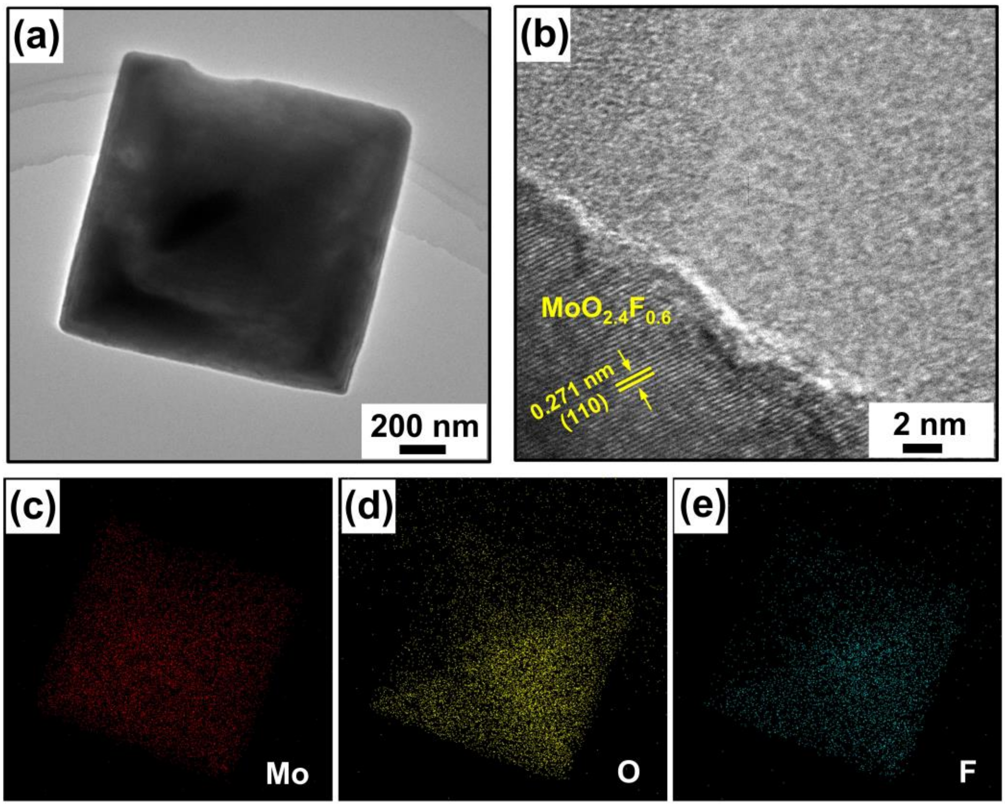

Figure S18. (a) TEM images, (b) HRTEM image, and (c-e) EDS mapping images of c-$MoO_{2.4}F_{0.6}$.

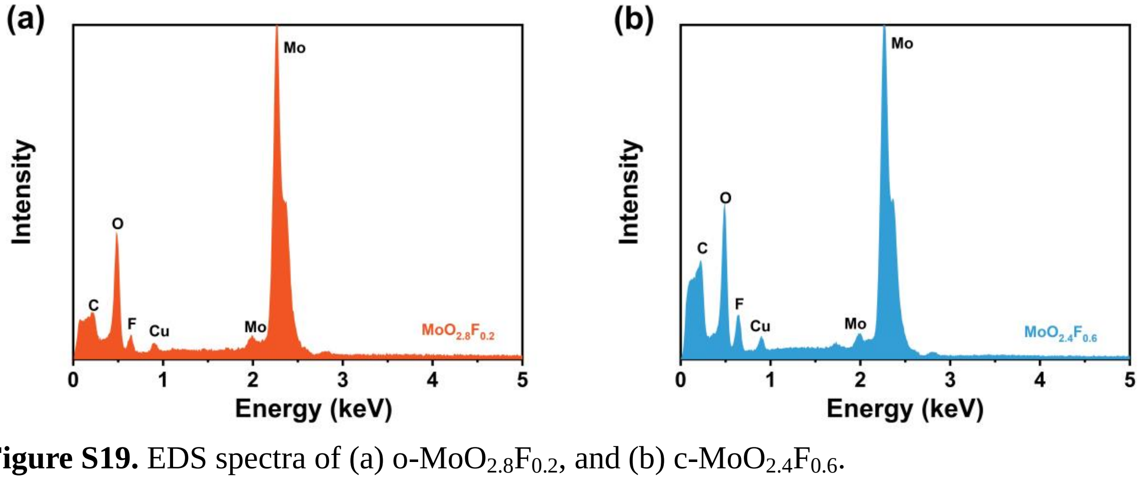

Figure S19. EDS spectra of (a) o-$MoO_{2.8}F_{0.2}$, and (b) c-$MoO_{2.4}F_{0.6}$.

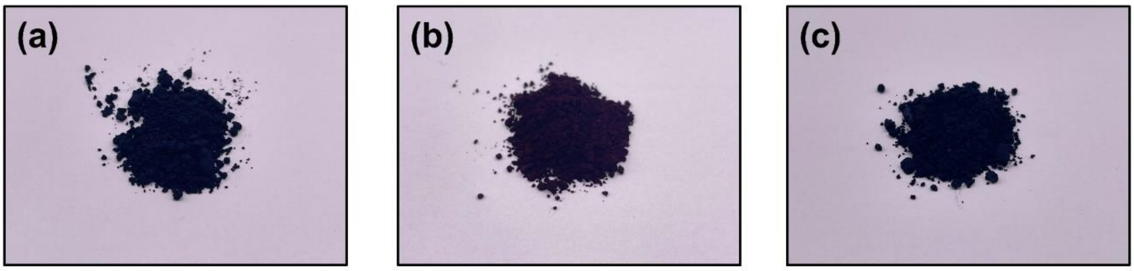

Figure S20. Photographs of (a) o-$MoO_{2.8}F_{0.2}$, (b) c-$MoO_{2.4}F_{0.6}$, and (c) o-c $MoO_{2.8}F_{0.2}/MoO_{2.4}F_{0.6}$ heterostructures.

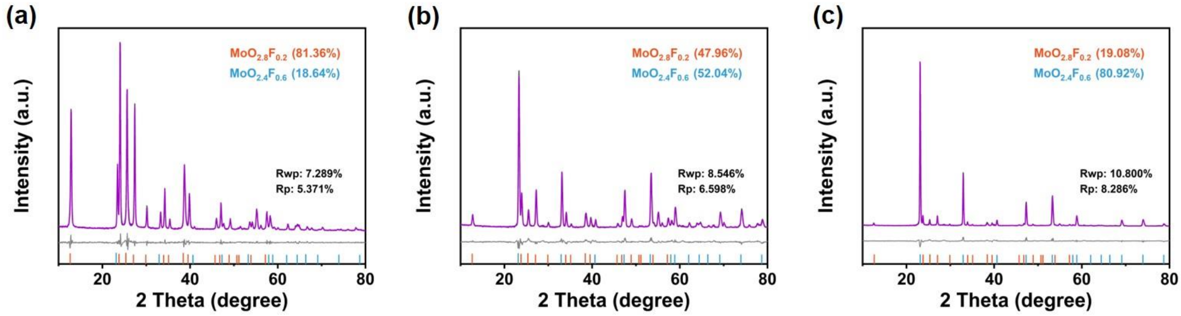

Figure S21. X-ray diffraction (XRD) patterns of o-c $MoO_{2.8}F_{0.2}/MoO_{2.4}F_{0.6}$ heterostructures with different phases ratio and their Rietveld refinement.

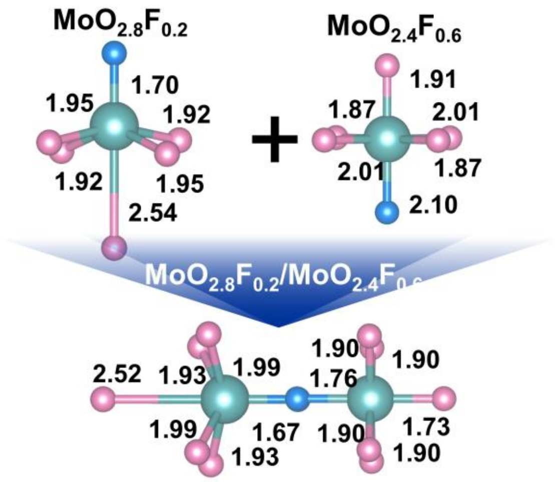

Figure S22. The coordination structure of Raman spectra.

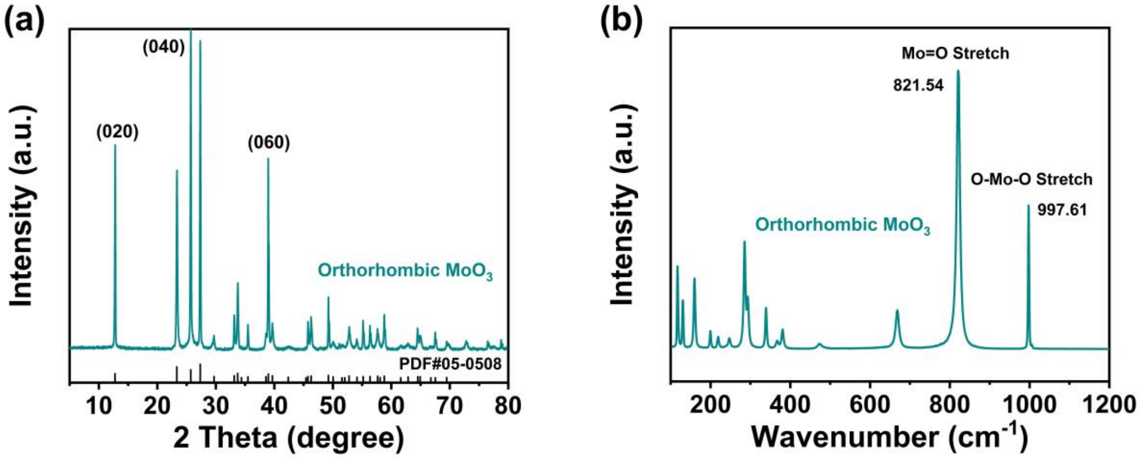

Figure S23. (a) XRD patterns and (b) Raman spectrum of MoO_3.

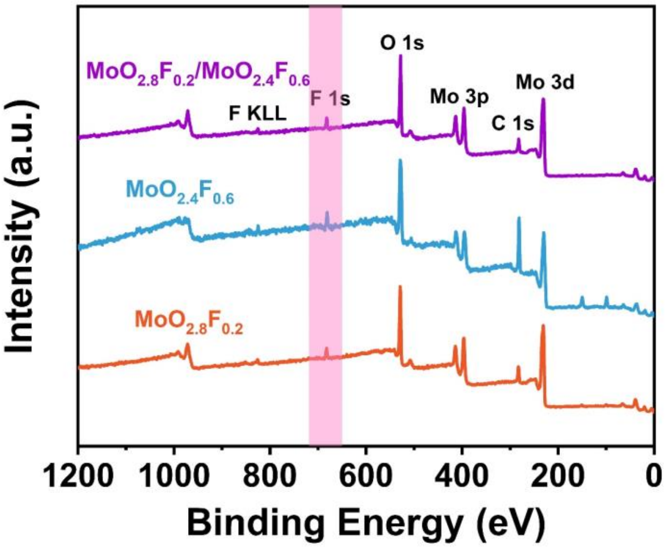

Figure S24. XPS survey spectra of o-c $MoO_{2.8}F_{0.2}/MoO_{2.4}F_{0.6}$ heterostructures.

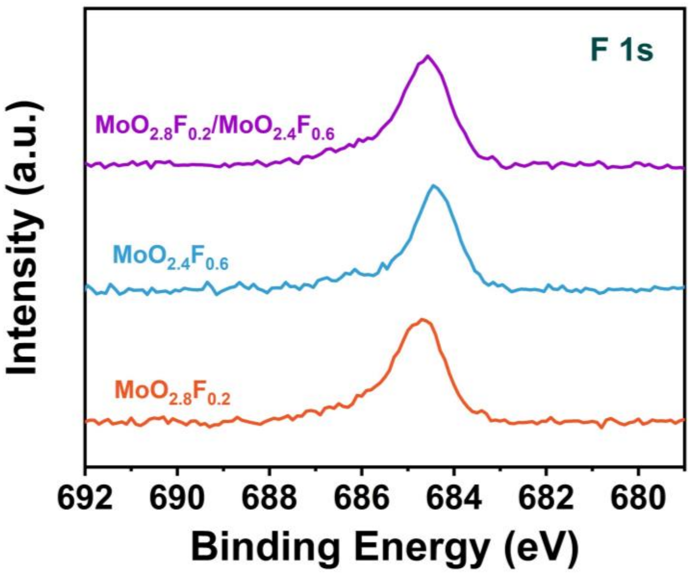

Figure S25. F 1s XPS spectra of o-$MoO_{2.8}F_{0.2}$, c-$MoO_{2.4}F_{0.6}$ and o-c $MoO_{2.4}F_{0.6}/MoO_{2.8}F_{0.2}$ heterostructures.

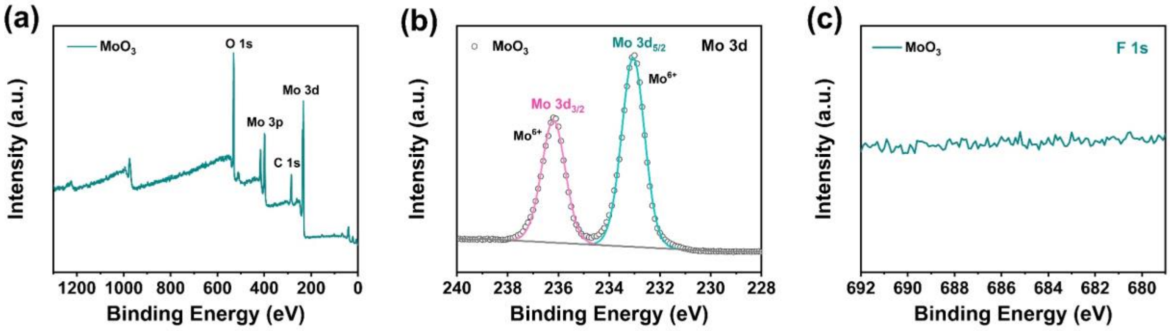

Figure S26. (a) XPS survey spectra, (b) Mo 3d XPS spectra, and (c) F 1s XPS spectra of MoO_3.

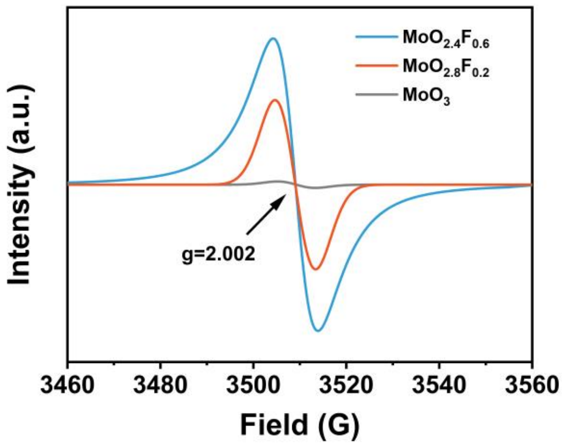

Figure S27. EPR spectra of the MoO_3, $MoO_{2.8}F_{0.2}$ and $MoO_{2.4}F_{0.6}$ samples at room temperature.

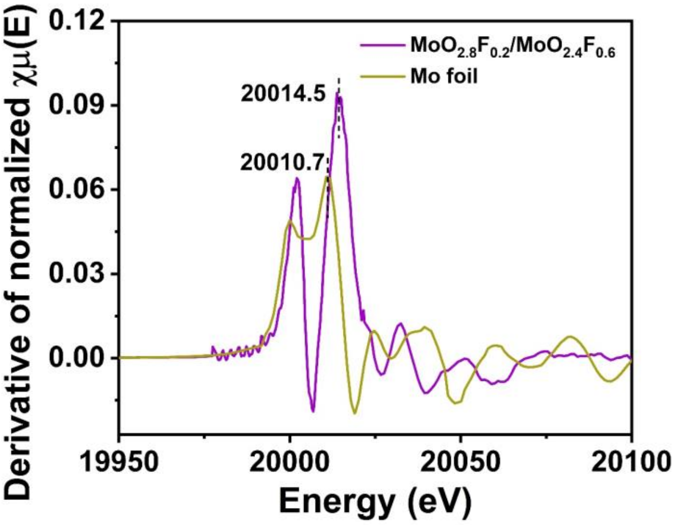

Figure S28. The derivative XANES curves of the Mo foil and o-c $MoO_{2.8}F_{0.2}/MoO_{2.4}F_{0.6}$ heterostructures at Mo *K*-edge.

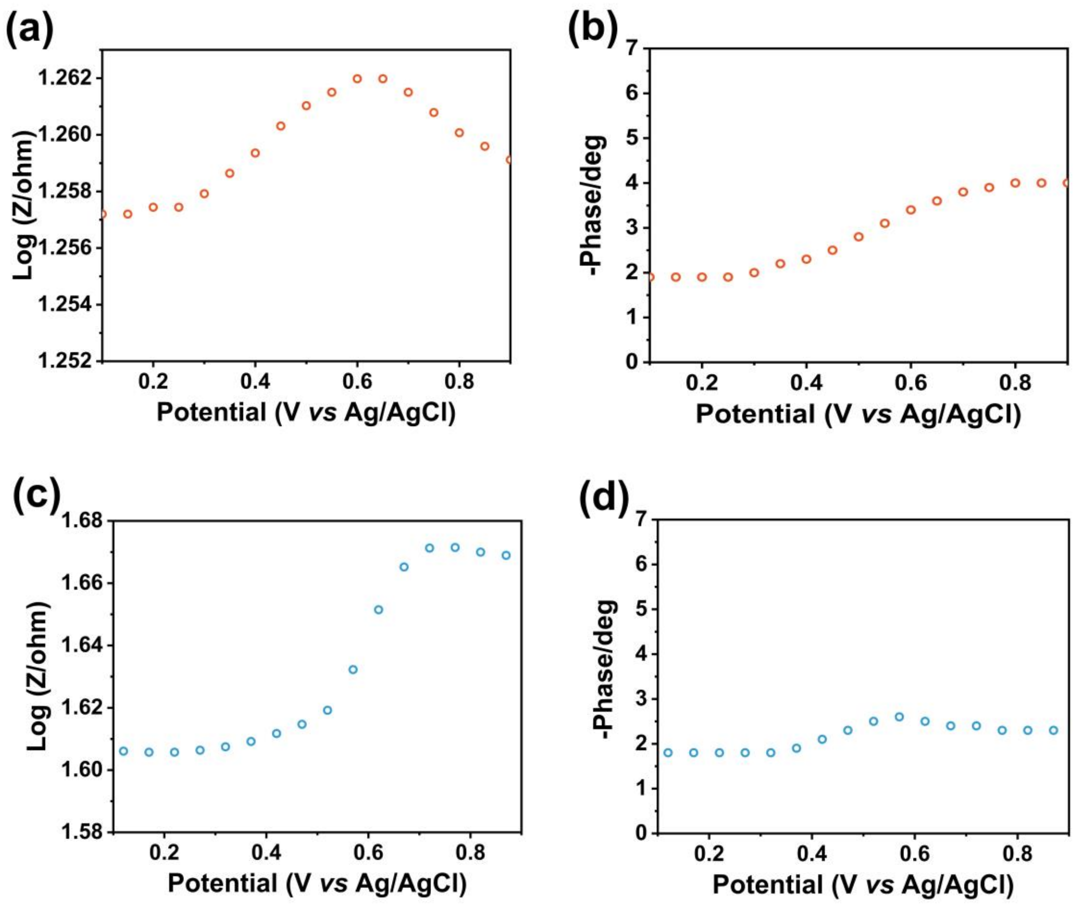

Figure S29. Mott-Schottky curves of (a, b) o-$MoO_{2.8}F_{0.2}$, and (c, d) c-$MoO_{2.4}F_{0.6}$.

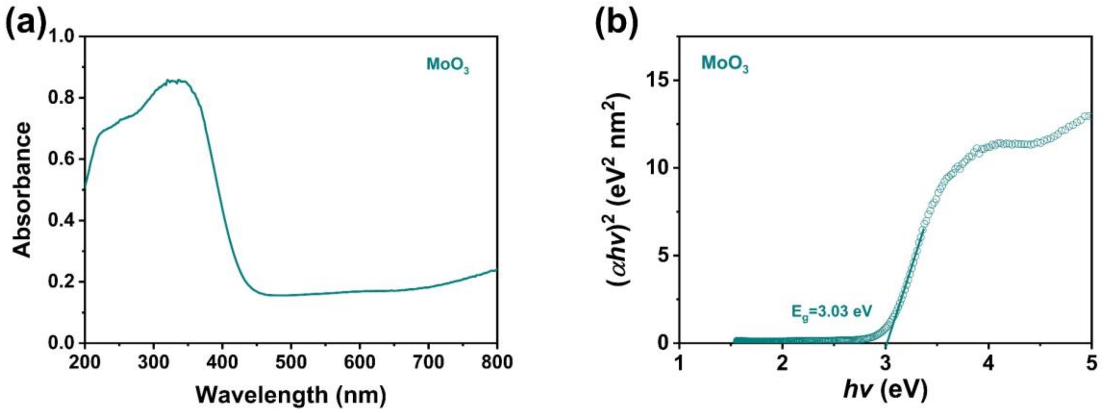

Figure S30. (a) UV−vis diffuse reflectance spectra, and (b) photon energy vs $(\alpha hv)^2$ curves of MoO_3.

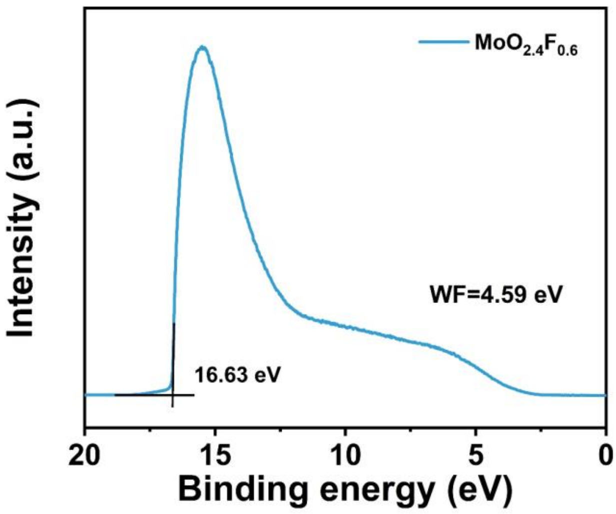

Figure S31. UPS curves of the c-$MoO_{2.4}F_{0.6}$.

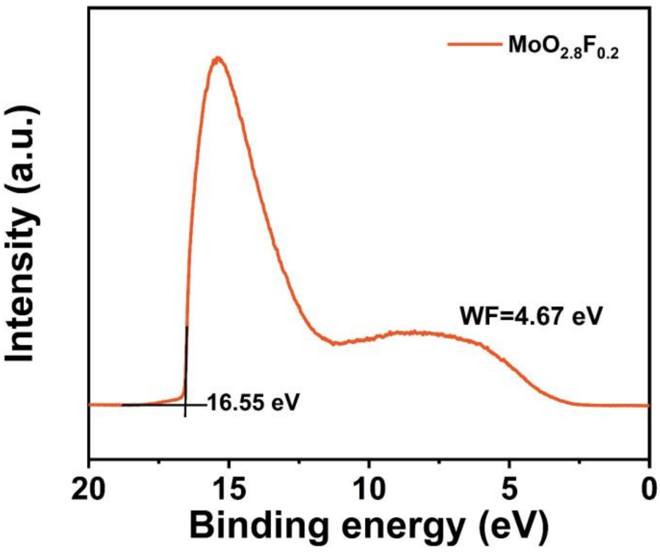

Figure S32. UPS curves of the o-$MoO_{2.8}F_{0.2}$.

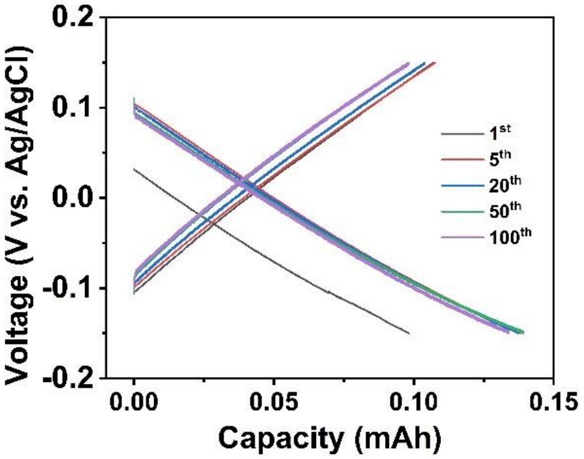

Figure R33. GCD curves of AC electrode in the three electrodes system.

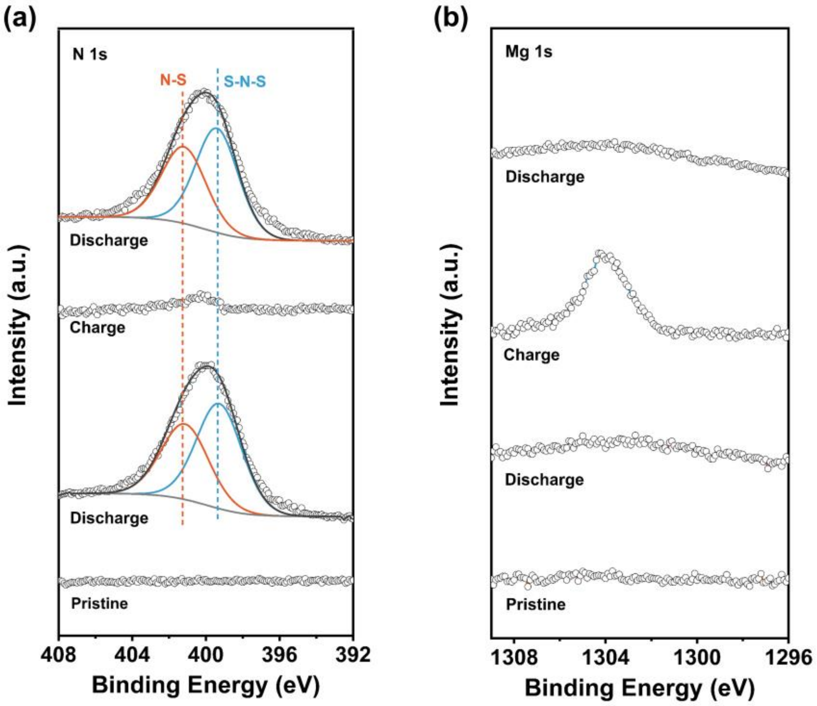

Figure R34. The ex-situ (a) N 1s XPS spectra, (b) Mg 1s XPS spectra of the AC electrode at discharged and charged states.

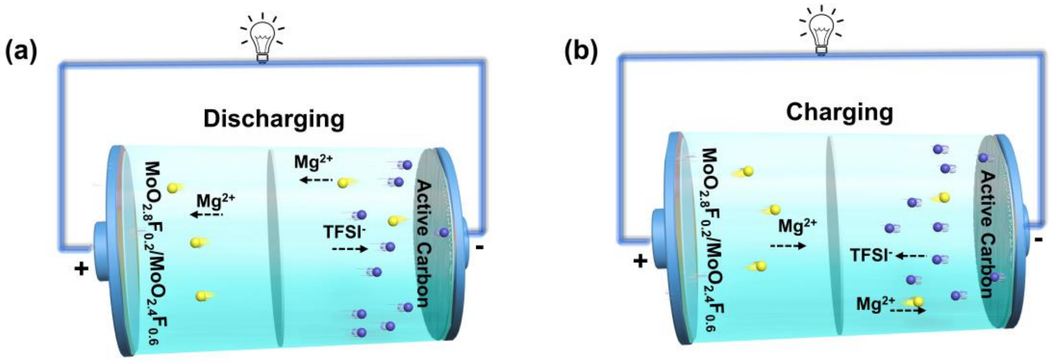

Figure R35. (a, b) Schematic illustration of the discharge/charge processes of the $MoO_{2.8}F_{0.2}/MoO_{2.4}F_{0.6}$//AC magnesium battery.

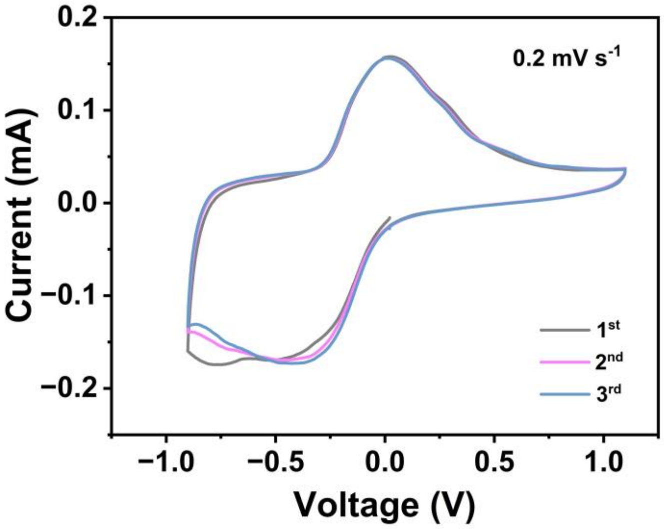

Figure S36. CV curves at 0.2 mV s^{-1} of o-c MoO2.8F0.2/MoO2.4F0.6 electrode.

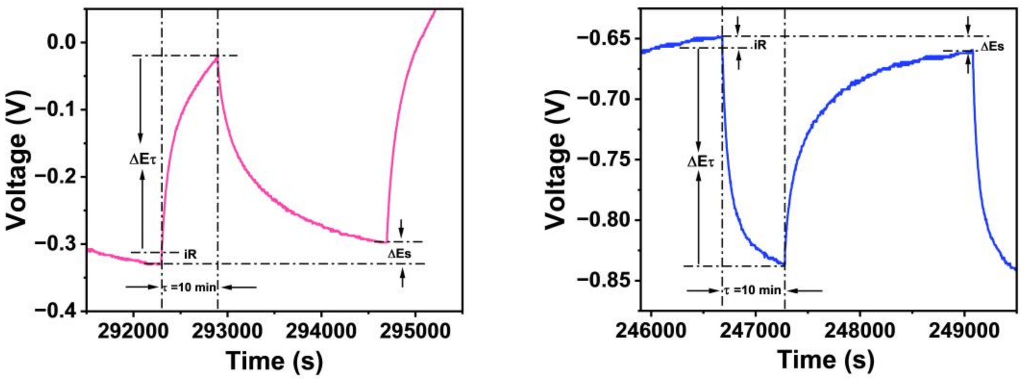

Figure S37. E versus t curves of o-c $MoO_{2.8}F_{0.2}$/$MoO_{2.4}F_{0.6}$ electrode for a single GITT during charge process and discharge process.

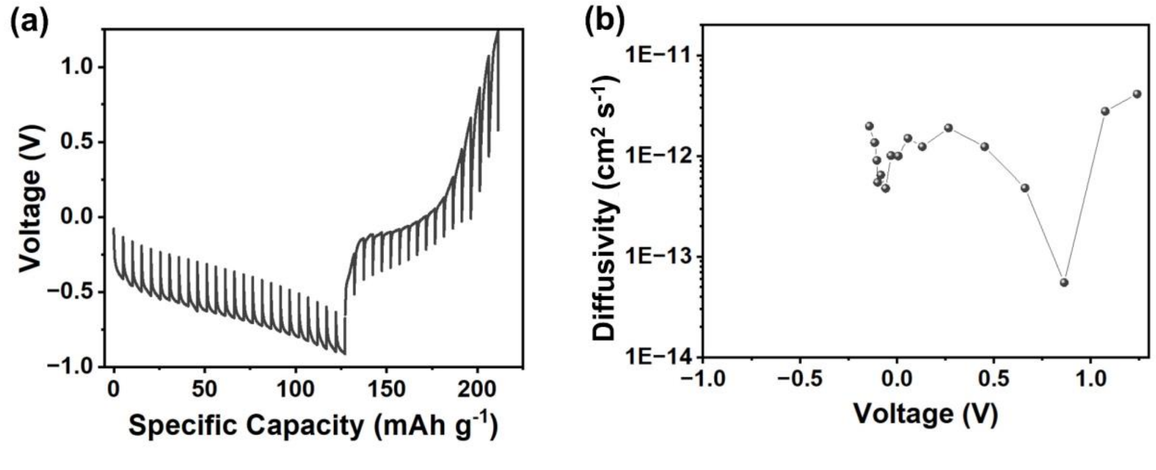

Figure S38. (a) GITT curve, and (b) calculated Mg^{2+} diffusion coefficients of c-$MoO_{2.4}F_{0.6}$ electrodes.

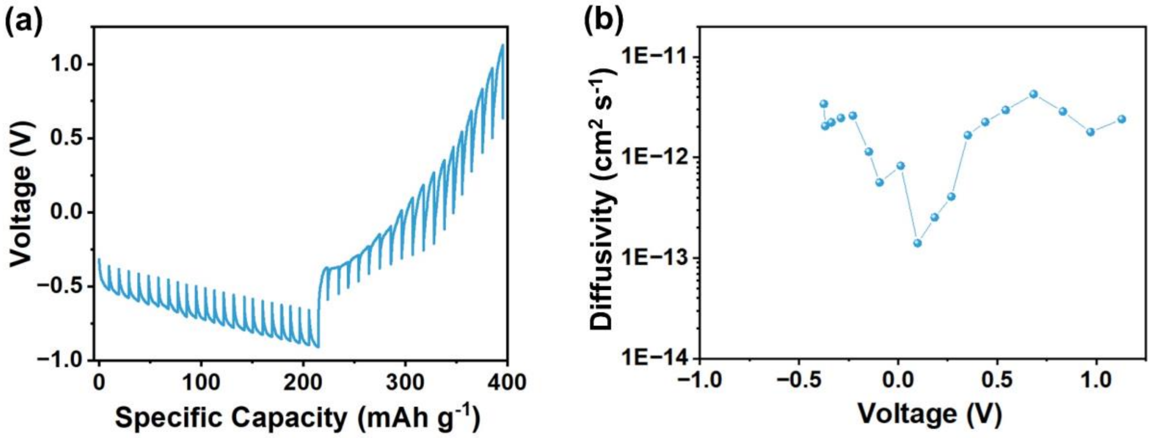

Figure S39. (a) GITT curve, and (b) calculated Mg^{2+} diffusion coefficients of o-$MoO_{2.8}F_{0.2}$ electrodes.

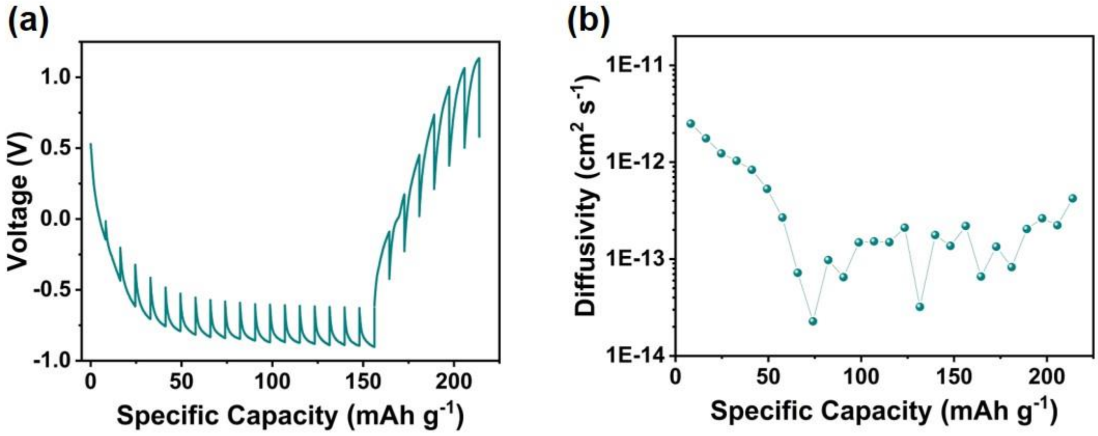

Figure S40. (a) GITT curve, and (b) calculated Mg^{2+} diffusion coefficients of MoO_3 electrode.

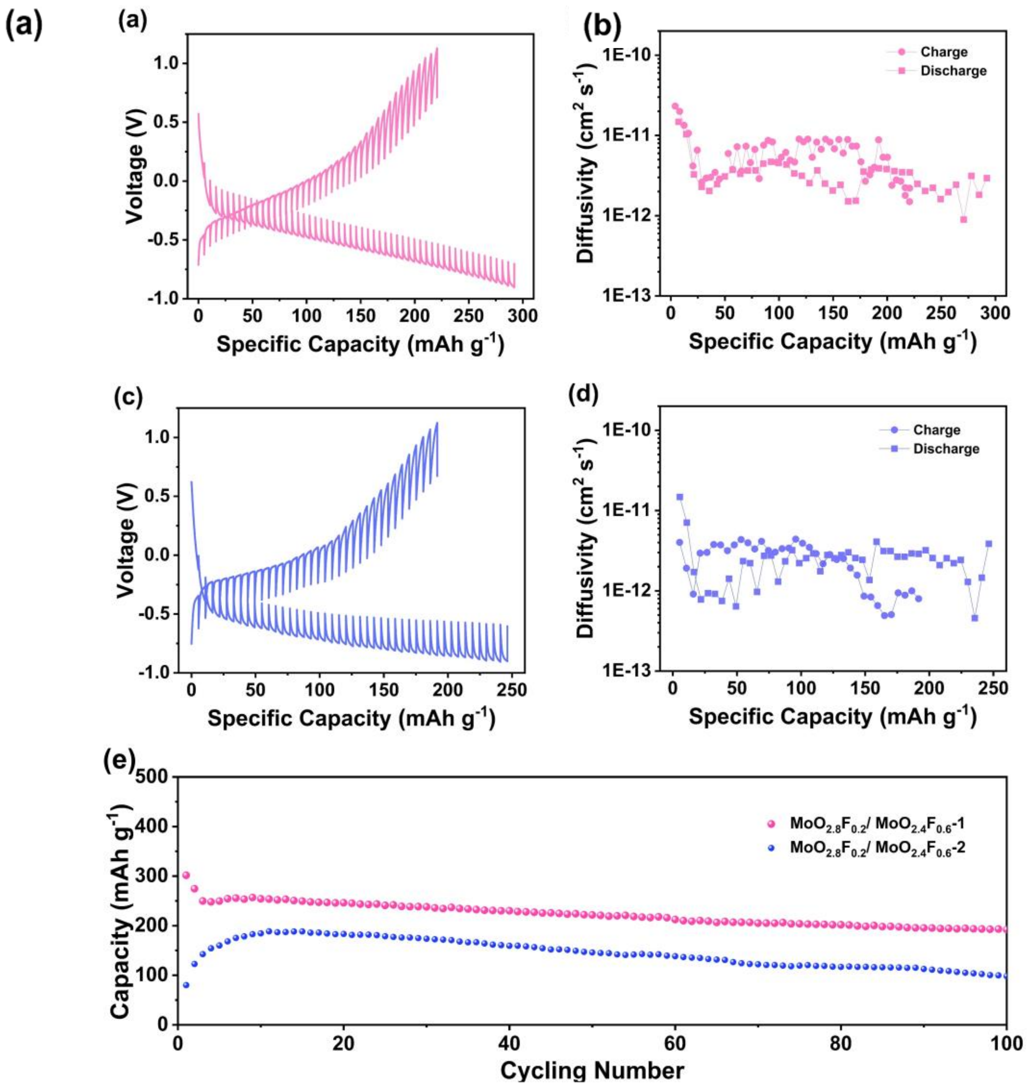

Figure S41. (a, c) GITT curve, and (b, d) calculated Mg^{2+} diffusion coefficients of $MoO_{2.8}F_{0.2}$/ $MoO_{2.4}F_{0.6}$-1 and $MoO_{2.8}F_{0.2}$/ $MoO_{2.4}F_{0.6}$-2 electrode. (e) Cycling performances at 0.1 A g^{-1} of $MoO_{2.8}F_{0.2}$/ $MoO_{2.4}F_{0.6}$-1 and $MoO_{2.8}F_{0.2}$/ $MoO_{2.4}F_{0.6}$-2 electrodes.

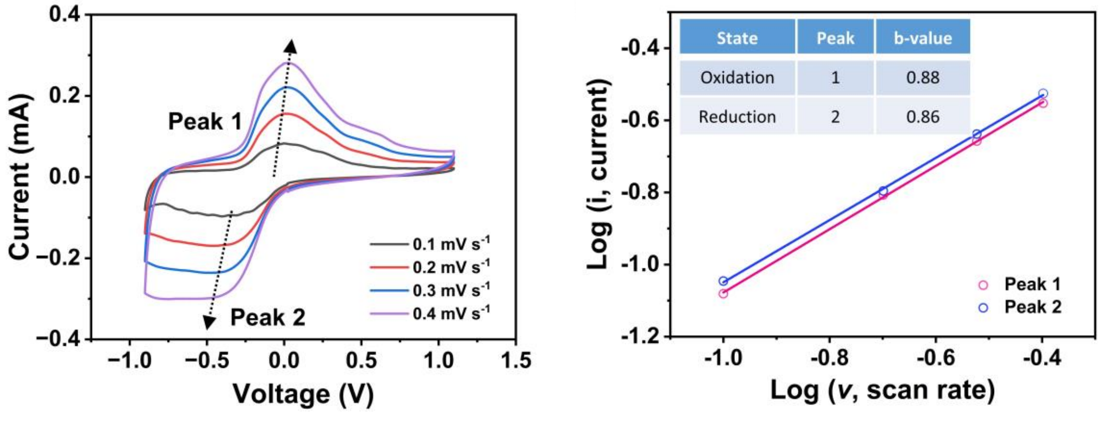

Figure S42. (a) CV curves at the different scan rates, and (b) relationship between peak current and scan rates of o-c $MoO_{2.8}F_{0.2}/MoO_{2.4}F_{0.6}$ electrode.

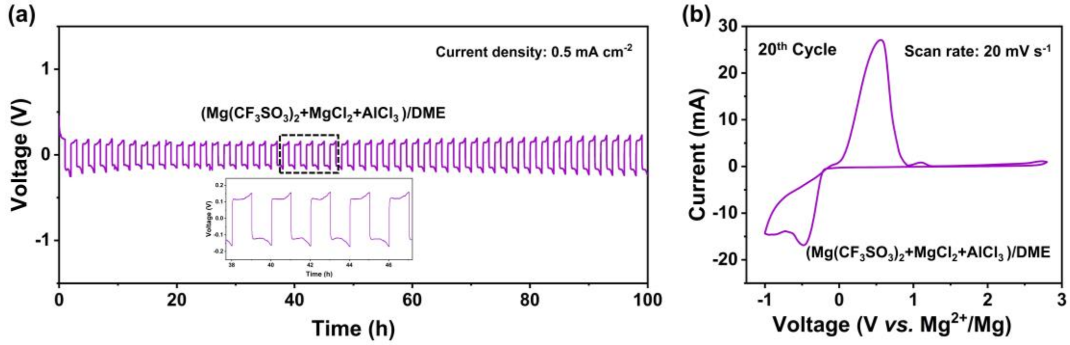

Figure S43. (a) Galvanostatic Mg plating/stripping in Mg||Mg symmetric cells at 0.5 mA cm^{-2}. (b) CV curve of the Mg plating and stripping process using $Mg(CF_3SO_3)_2$+$MgCl_2$+$AlCl_3$/DME electrolyte.

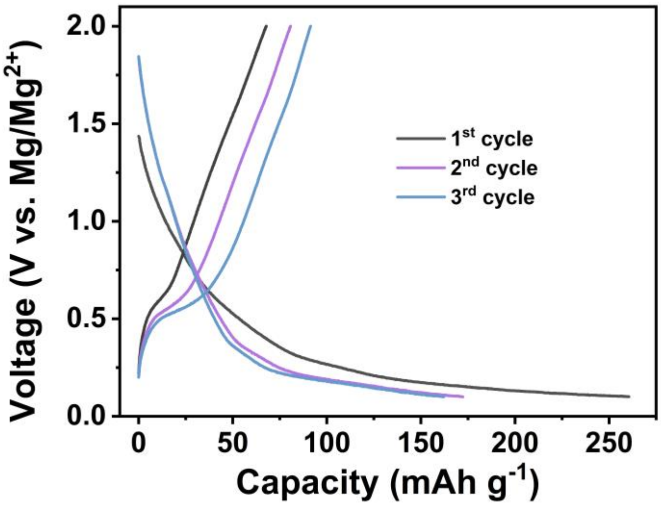

Figure S44. The GCD profiles of the $MoO_{2.8}F_{0.2}$/$MoO_{2.4}F_{0.6}$//Mg full cell.

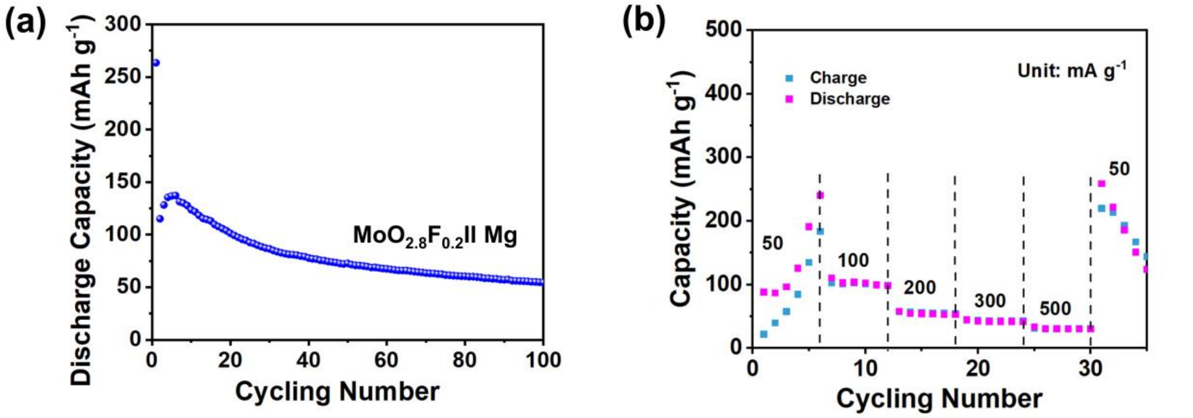

Figure S45. (a) Cycling performances at 0.1 A g^{-1}, and (b) rate capability at 0.05–0.5 A g^{-1} of o-$MoO_{2.8}F_{0.2}$ electrodes.

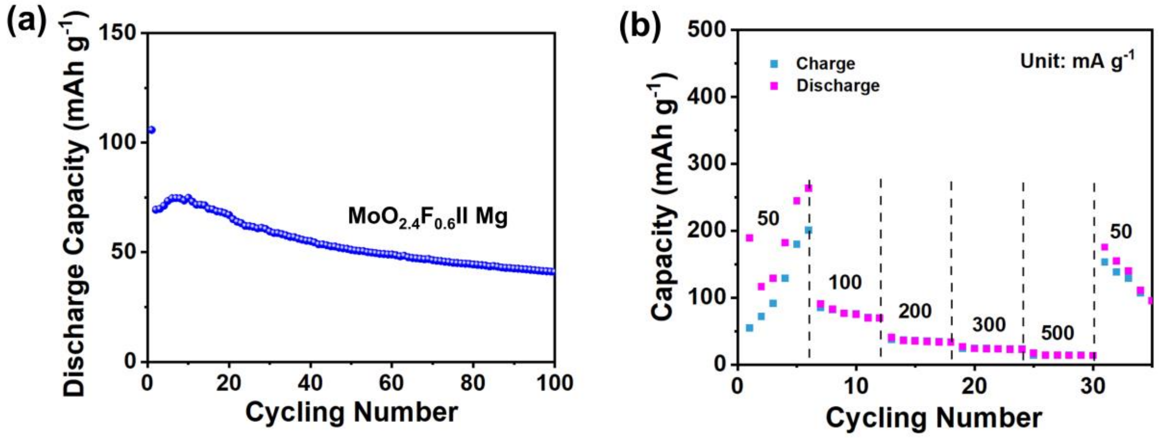

Figure S46. (a) Cycling performances at 0.1 A g^{-1}, and (b) rate capability at 0.05–0.5 A g^{-1} of c-$MoO_{2.4}F_{0.6}$ electrodes.

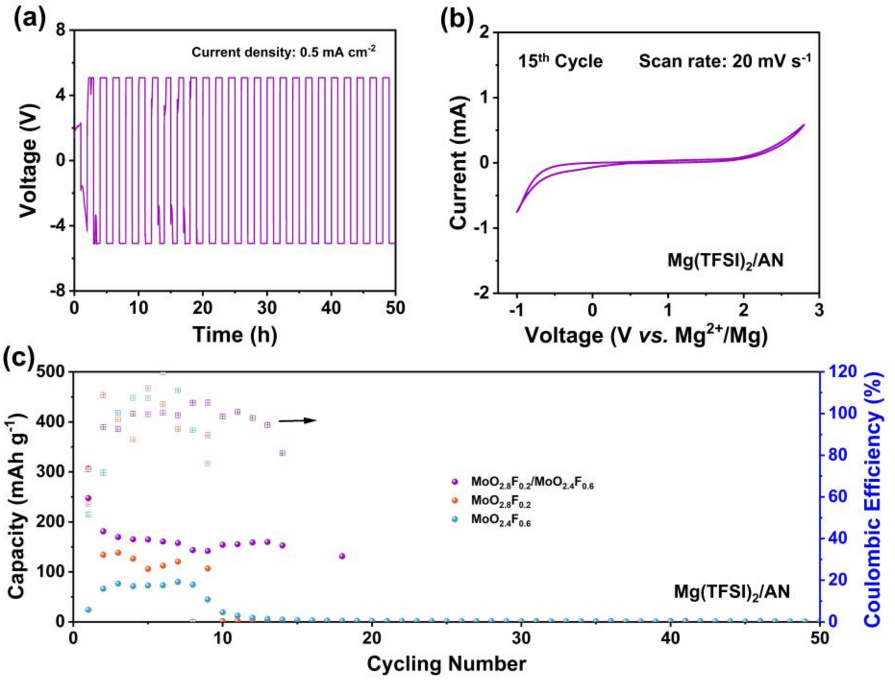

Figure R47. (a) Galvanostatic Mg plating/stripping in Mg||Mg symmetric cells at 0.5 mA cm^{-2}. (b) CV curve of the Mg plating and stripping process using $Mg(TFSI)_2$/AN electrolyte. (c) Cycling performances at 0.1 A g^{-1} of $MoO_{2.8}F_{0.2}/MoO_{2.4}F_{0.6}$//Mg, $MoO_{2.8}F_{0.2}$//Mg, and $MoO_{2.4}F_{0.6}$//Mg in $Mg(TFSI)_2$/AN electrolyte.

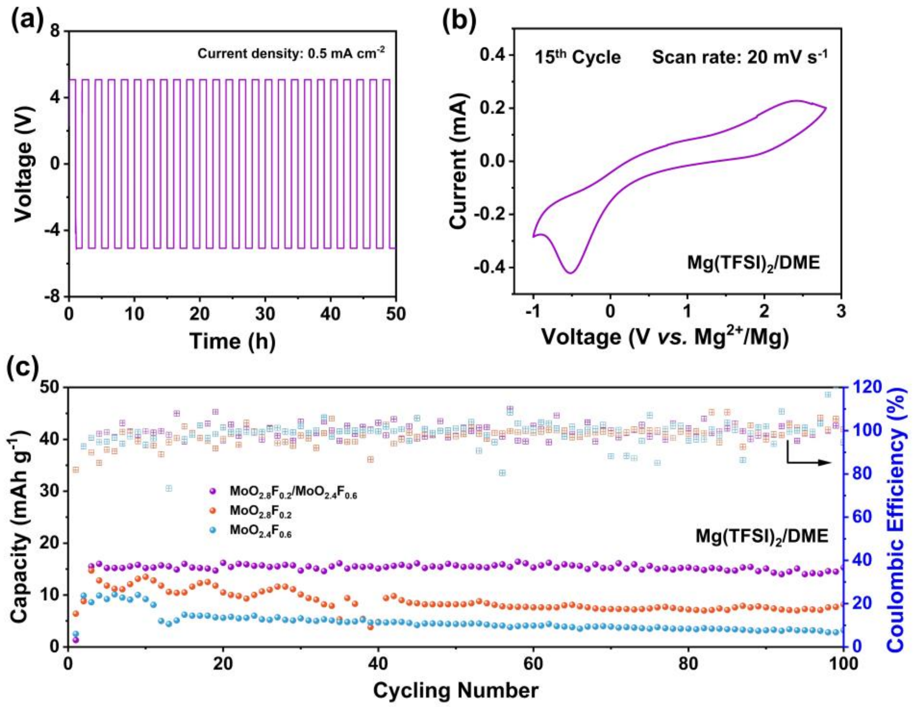

Figure R48. (a) Galvanostatic Mg plating/stripping in Mg||Mg symmetric cells at 0.5 mA cm^{-2}. (b) CV curve of the Mg plating and stripping process using $Mg(TFSI)_2$/DME electrolyte. (c) Cycling performances at 0.1 A g^{-1} of $MoO_{2.8}F_{0.2}/MoO_{2.4}F_{0.6}$//Mg, $MoO_{2.8}F_{0.2}$//Mg, and $MoO_{2.4}F_{0.6}$//Mg in $Mg(TFSI)_2$/DME electrolyte.

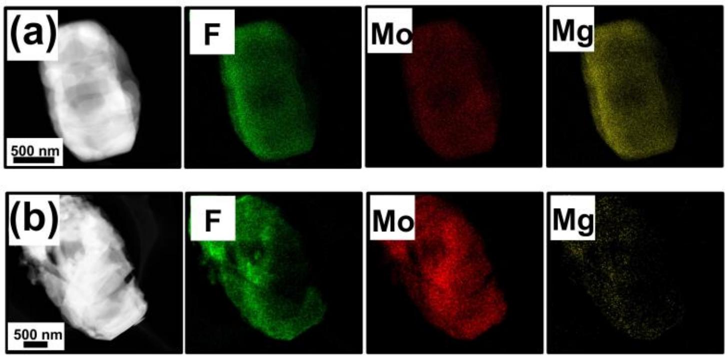

Figure S49. The ex-situ EDS mapping images of the o-c $MoO_{2.8}F_{0.2}$/$MoO_{2.4}F_{0.6}$ electrode at various discharged/charged states.

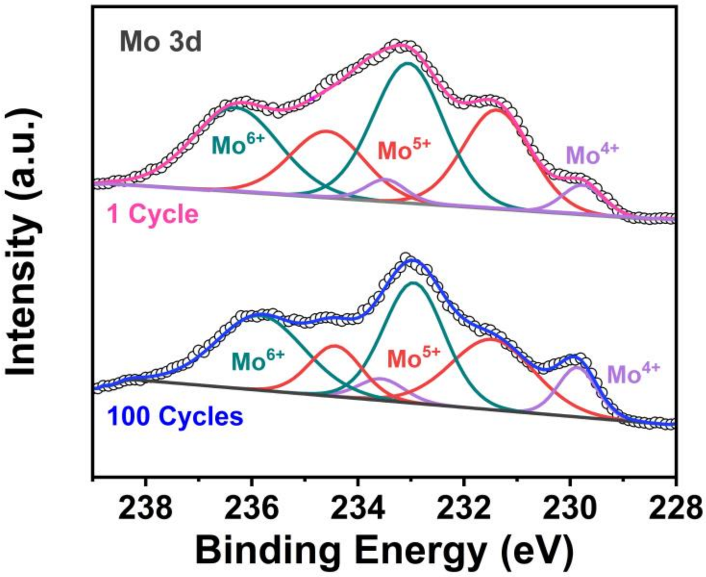

Figure S50. Mo 3d XPS spectra of o-c $MoO_{2.8}F_{0.2}$/$MoO_{2.4}F_{0.6}$ electrode after 100 cycles.

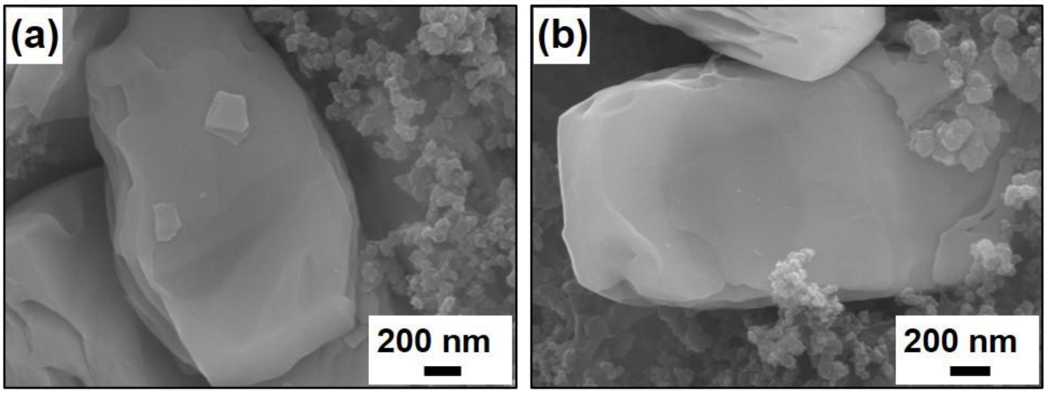

Figure S51. SEM images of dual-phase $MoO_{2.8}F_{0.2}/MoO_{2.4}F_{0.6}$ heterostructure after 100 cycles.

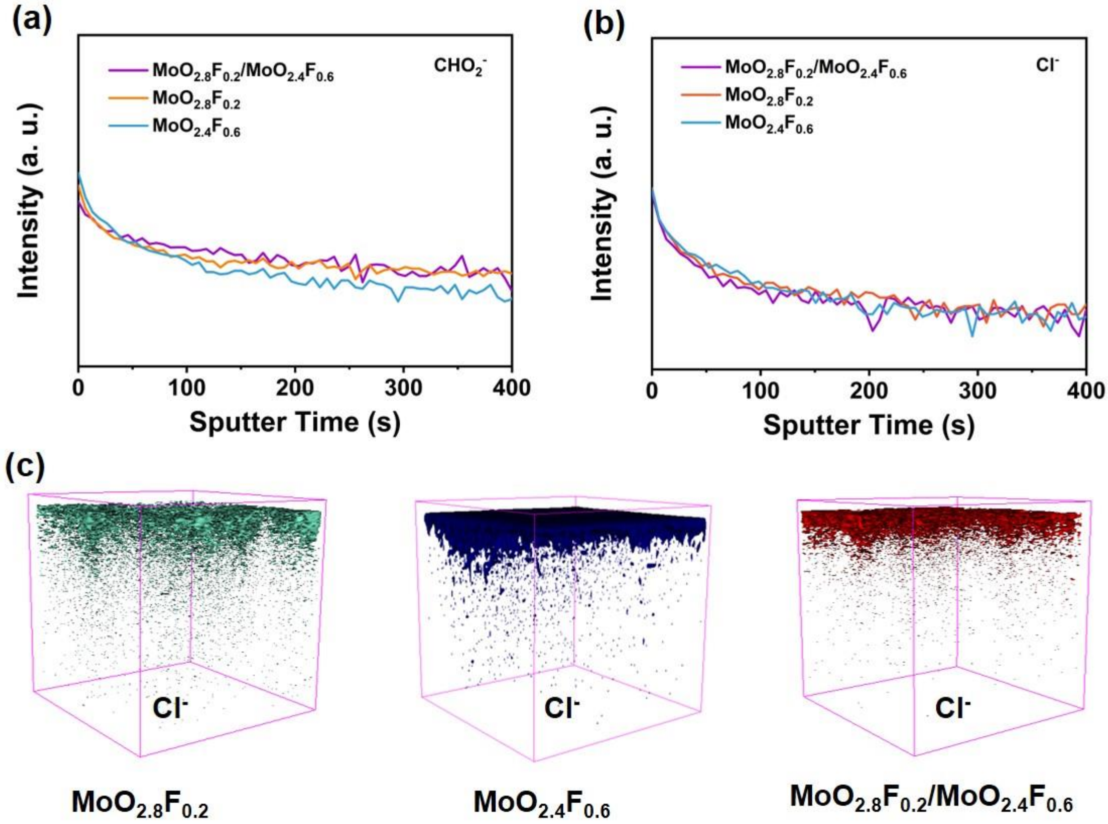

Figure S52. (a, b) The TOF-SIMS depth profiles of electrolyte decomposition species in $MoO_{2.8}F_{0.2}$, $MoO_{2.4}F_{0.6}$ and $MoO_{2.8}F_{0.2}/MoO_{2.4}F_{0.6}$ cathodes. (c) The TOF-SIMS 3D views of Cl^- ionic fragments in $MoO_{2.8}F_{0.2}$, $MoO_{2.4}F_{0.6}$ and $MoO_{2.8}F_{0.2}/MoO_{2.4}F_{0.6}$ cathodes.

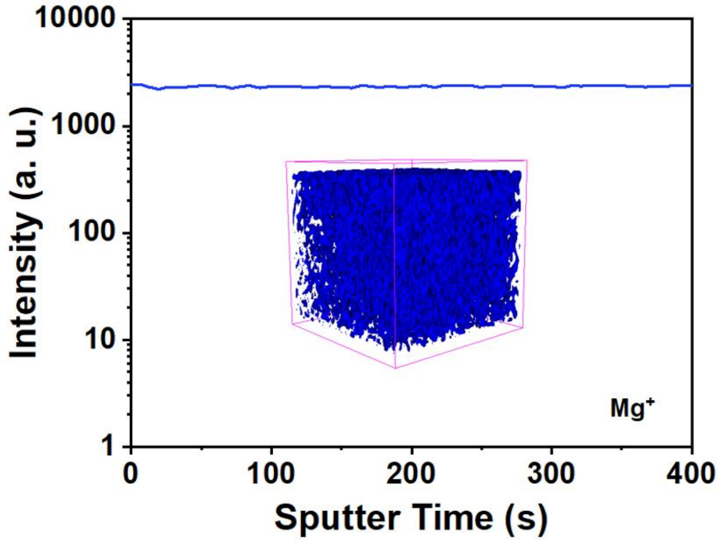

Figure R53. The TOF-SIMS 3D views and depth profiles of the Mg^+ ionic fragments in o-c $MoO_{2.8}F_{0.2}/MoO_{2.4}F_{0.6}$ cathodes.

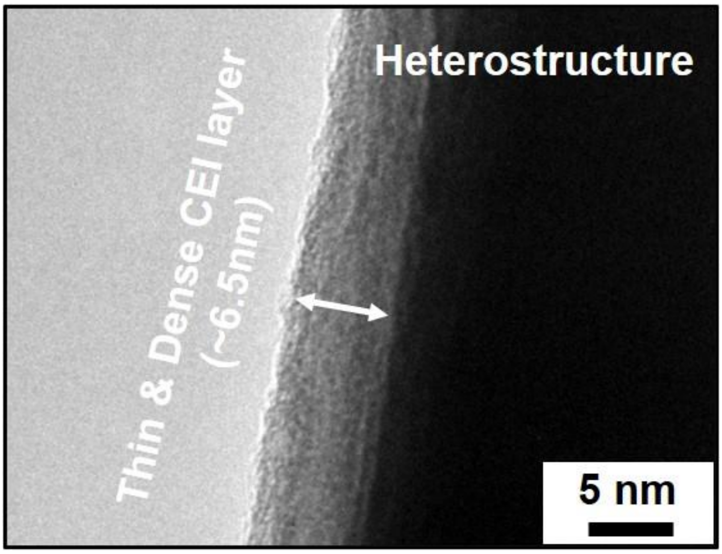

Figure S54. TEM images of $MoO_{2.8}F_{0.2}/MoO_{2.4}F_{0.6}$ cathodes after 20 cycles.

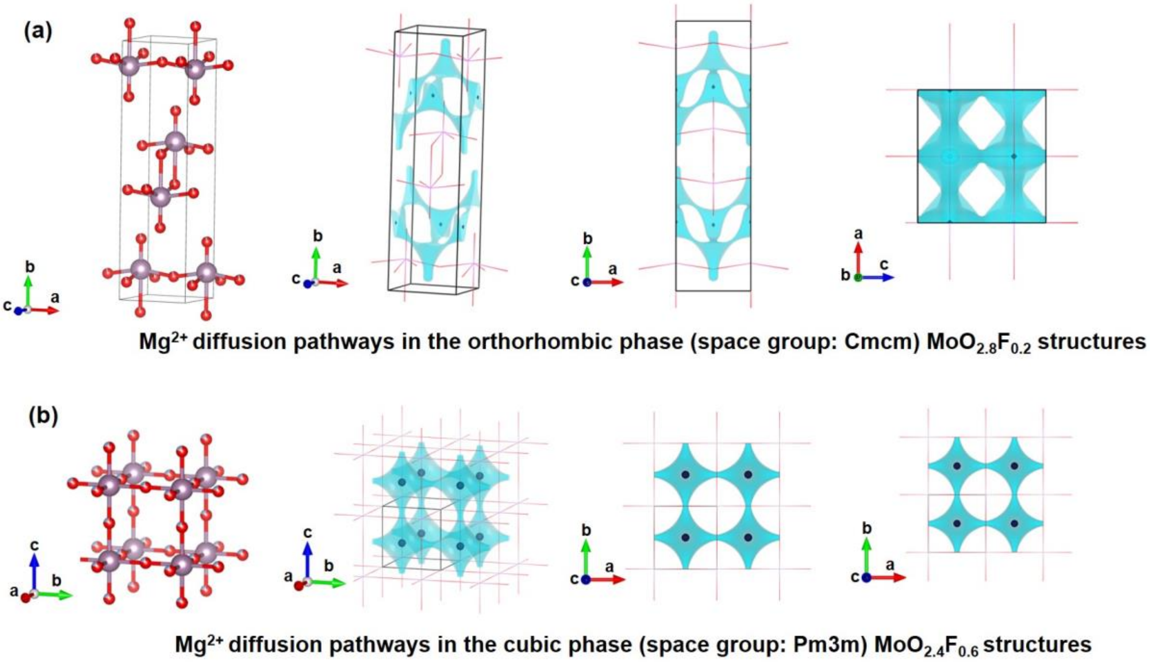

Figure S55. The Mg^{2+} migration channels (blue isosurface) revealed by BVSE calculation for o-$MoO_{2.8}F_{0.2}$ and c-$MoO_{2.4}F_{0.6}$ crystals, respectively.

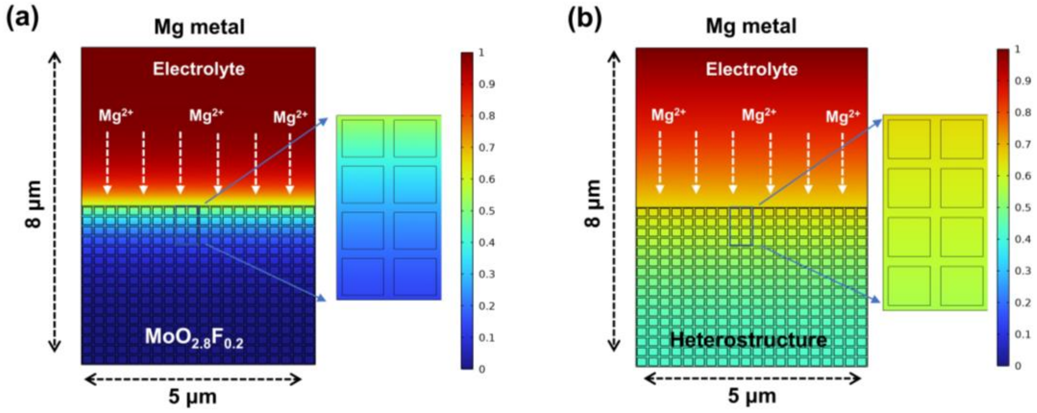

Figure S56. Finite element simulation of Mg^{2+} concentration distribution for (a) o-$MoO_{2.8}F_{0.2}$ and (b) o-c $MoO_{2.8}F_{0.2}/MoO_{2.4}F_{0.6}$ heterostructures.

Table S1. Experimental Raman modes of MoO_3 and band assignment.

Raman shift (cm^{-1})	Assignment
109.0	B_{2g}, translational rigid MoX_4 chain mode, T_c
120.1	B_{3g}, translational rigid MoX_4 chain mode, T_c
140.9	A_g/B_{1g}, translational rigid MoX_4 chain mode, T_b
279.6	B_{3g}, δ X=Mo=X wagging
328.0/335.1	A_g, B_{1g}, δ X–Mo–X bend
376.4/ 393.2	B_{1g}, δ X–Mo–X scissor
444.3	Mo–X–Mo deformation
663.3	B_{2g}, B_{3g}, ν_{as} X–Mo–X stretch
813.1/ 845.7	A_g, ν_s Mo=X stretch
991.3/1002.7	A_g, ν_{as} Mo=X stretch

Table S2. Optical properties of o-$MoO_{2.8}F_{0.2}$ and c-$MoO_{2.4}F_{0.6}$.

Sample	E_{fb} (eV)	E_g (eV)	E_{CB} (eV)	E_{VB} (eV)
$MoO_{2.8}F_{0.2}$	0.43	1.79	0.23	2.02
$MoO_{2.6}F_{0.4}$	0.39	1.56	0.19	1.75

Table S3. Ion diffusion coefficients of recently reported molybdenum-based electrode materials for Li-ion battery and other electrode materials for Mg-ion battery.

Materials	D (cm^2 s^{-1})	Battery type	Reference
MoO_{3-x} /MXene heterostructure	$10^{-14} - 10^{-12}$	Li	5
α-$Mo(O/F)_3$	$10^{-16} - 10^{-12}$	Li	6
3% Mo-VS_4	$1.10 \times 10^{-12} - 15.70 \times 10^{-12}$	Mg	7
Mo-VS_4/N-TG	$6.60 \times 10^{-13} - 2.63 \times 10^{-11}$	Mg	8
HMoOF	$2.32 \times 10^{-11} - 2.89 \times 10^{-13}$	Mg	9
MoS_2–PEO	$10^{-13} - 10^{-11}$	Mg	10
$MoO_{2.8}F_{0.2}$/$MoO_{2.6}F_{0.4}$ heterostructure	$9.12 \times 10^{-13} - 1.99 \times 10^{-10}$	Mg	**This work**

Table S4. Electrochemical performance of MoO_3-based electrodes for polyvalent ion battery.

Electrode	Electrolyte	Specific capacity	Capacity retention	Refe-rence
$MoO_{2.8}F_{0.2}$	0.2 M $Mg(TFSI)_2$ in propylene carbonate	~ 42 mA h g^{-1}	~178% / 18 cycles	11
MoO_3 thin-film	0.1 M $Mg(TFSI)_2$ in acetonitrile	220 mAh g^{-1} (0.3 μA cm^{-2})	95%/10 cycles (0.3 μA cm^{-2})	12
HMoOF	0.3 M $Mg(TFSI)_2$ in acetonitrile	241 mAh g^{-1} (100 mA g^{-1})	98%/800 cycles (2000 mA g^{-1})	9
Mo-VS_4/N-TG	0.4 M 2PhMgCl-$AlCl_3$ in tetrahydrofuran solution (APC/THF)	140 mAh g^{-1} (50 mA g^{-1})	80.6%/1200 cycle (500 mA g^{-1})	13

Cu_2MoS_4	G_4-HDMS	180 mAh g^{-1} (50 mA g^{-1})	60%/2500 cycle (500 mA g^{-1})	14
α-MoO_3	1 M $Mg(ClO_4)_2$/AN+ 3 mol% H_2O	210 mAh g^{-1} (20 uV s^{-1})	–	15
$Mg_2Mo_3O_8$	0.5 M $[Mg(G_4)][TFSA]_2$/ $[PYR_{13}][TFSA]$	89 mAh g^{-1} (5 mA g^{-1})	–	16
V_2MoO_8	APC/1.0 M LiCl	187.4 mAh g^{-1} (20 mA g^{-1})	72.5%/50th cycle 20 mA g^{-1}	17
$MoO_{2.8}F_{0.2}$/ $MoO_{2.4}F_{0.6}$ heterostructure	0.3 M $Mg(TFSI)_2$ in acetonitrile	303.8 mAh g^{-1} (100 mA g^{-1})	78.5%/600 cycles (1000 mA g^{-1})	**This work**
$MoO_{2.8}F_{0.2}$/ $MoO_{2.4}F_{0.6}$ //Mg Cell	0.2M $Mg(CF_3SO_3)_2$ +0.4 M of $MgCl_2$ +0.4 M $AlCl_3$ in 1,2-dimethoxyethane	172.5 mAh g^{-1} (100 mA g^{-1})		**This work**

REFERENCES

1. Kresse G, and Joubert D. From ultrasoft pseudopotentials to the projector augmented-wave method. *Phys Rev B* 1999; **59**: 1758.
2. Perdew J, Burke K and Ernzerhof M. Generalized gradient approximation made simple. *Phys Rev Lett* 1996; **77**: 3865.
3. Grimme S, Antony J and Ehrlich *et al.* A consistent and accurate ab initio parametrization of density functional dispersion correction (DFT-D) for the 94 elements H-Pu. *J Chem Phys* 2010; **132**: 154104.
4. Apergi S, Koch C and Brocks G *et al.* Decomposition of organic perovskite precursors on MoO_3: role of halogen and surface defects. *ACS Appl Mater Interfaces* 2022; **14**: 34208-19.
5. Zhang Y, Chen P and Wang Q *et al.* High‐capacity and kinetically accelerated lithium storage in MoO_3 enabled by oxygen vacancies and heterostructure. *Adv Energy Mater* 2021; **11**: 2101712.

6. Wustrow A, Hancock J and Incorvati J *et al.* Effect of fluoride doping on lithium diffusivity in layered molybdenum oxide. *ACS Appl Energy Mater* 2019; **2**: 2080-6.
7. Ding S, Li Z and Dai X *et al.* Mo-doped VS_4 with interlayer-expanded and engineering sulfur vacancies as cathode for advanced magnesium storage. *Chem Eng J* 2021; **417**: 129328.
8. Ding S, Dai X and Tian Y *et al.* Synergy strategy of electrical conductivity enhancement and vacancy introduction for improving the performance of VS_4 magnesium-ion battery cathode. *ACS Appl Mater Interfaces* 2021; **13**: 54005-17.
9. Wang W, Jiang Y and Yang Y *et al.* Basal planes unlocking and interlayer engineering endows proton doped-$MoO_{2.8}F_{0.2}$ with fast and stable magnesium storage. *ACS Nano* 2022; **16**: 17097-106.
10. Liang Y, Yoo H and Li Y *et al.* Interlayer-expanded molybdenum disulfide nanocomposites for electrochemical magnesium storage. *Nano Lett* 2015; **15**: 2194-202.
11. Incorvati J, Wan L and Key B *et al.* Reversible magnesium intercalation into a layered oxyfluoride cathode. *Chem Mater* 2016; **28**: 17-20.
12. Gershinsky G, Yoo H and Gofer Y *et al.* Electrochemical and spectroscopic analysis of Mg^{2+} intercalation into thin film electrodes of layered oxides: V_2O_5 and MoO_3. *Langmuir* 2013; 29: 10964.
13. Ding S, Dai X and Tian Y *et al.* Synergy strategy of electrical conductivity enhancement and vacancy introduction for improving the performance of VS_4 magnesium-ion battery cathode. *ACS Appl Mater Interfaces* 2021; **13**: 54005-17.
14. Zhang Y, Li T and Cao S *et al.* Cu_2MoS_4 hollow nanocages with fast and stable Mg^{2+}-storage performance. *Chem Eng J* 2020; **387**: 124125.
15. Spahr M, Novák P and Haas O *et al.* Electrochemical insertion of lithium, sodium, and magnesium in molybdenum(VI) oxide. *J Power Sources* 1995; **54**: 346-51.
16. Ishida N, Nakamura Y and Mandai T *et al.* Synthesis, cathode property and crystal, electronic and local structures of $Mg_2Mo_3O_8$ as Mg rechargeable battery cathode material. *Solid State Ionics* 2020; **354**: 115413.
17. Miao X, Chen Z and Wang N *et al.* Electrospun V_2MoO_8 as a cathode material for rechargeable batteries with Mg metal anode. *Nano Energy* 2017; **34**: 26-35.
